# Supplementation Strategies for Strength and Power Athletes: Carbohydrate, Protein, and Amino Acid Ingestion

**DOI:** 10.3390/nu16121886

**Published:** 2024-06-14

**Authors:** Stephen P. Bird, Mitch Nienhuis, Brian Biagioli, Kevin De Pauw, Romain Meeusen

**Affiliations:** 1 School of Health and Medical Sciences, University of Southern Queensland, Ipswich, QLD 4305, Australia; 2 Centre for Health Research, University of Southern Queensland, Ipswich, QLD 4305, Australia; 3 Movement Science, Grand Valley State University, Allendale, MI 49401, USA; 4 Kinesiology and Sport Sciences, School of Education and Human Development, University of Miami, Coral Gables, FL 33146, USA; 5 Human Physiology and Sports Physiotherapy Research Group (MFYS), Vrije Universiteit Brussel, 1050 Brussel, Belgium; 6 Brussels Human Robotics Research Center (BruBotics), Vrije Universiteit Brussel, 1050 Brussel, Belgium; 7 Department of Sports, Recreation, Exercise and Sciences, University of the Western Cape, Cape Town 7535, South Africa

**Keywords:** athlete, strength, supplements, nutrition, carbohydrate, protein, amino acids

## Abstract

It is a common belief amongst strength and power athletes that nutritional supplementation strategies aid recovery by shifting the anabolic/catabolic profile toward anabolism. Factors such as nutrient quantity, nutrient quality, and nutrient timing significantly impact upon the effectiveness of nutritional strategies in optimizing the acute responses to resistance exercise and the adaptive response to resistance training (i.e., muscle growth and strength expression). Specifically, the aim of this review is to address carbohydrates (CHOs), protein (PRO), and/or amino acids (AAs) supplementation strategies, as there is growing evidence suggesting a link between nutrient signaling and the initiation of protein synthesis, muscle glycogen resynthesis, and the attenuation of myofibrillar protein degradation following resistance exercise. Collectively, the current scientific literature indicates that nutritional supplementation strategies utilizing CHO, PRO, and/or AA represents an important approach aimed at enhancing muscular responses for strength and power athletes, primarily increased muscular hypertrophy and enhanced strength expression. There appears to be a critical interaction between resistance exercise and nutrient–cell signaling associated with the principle of nutrient timing (i.e., pre-exercise, during, and post-exercise). Recommendations for nutritional supplementation strategies to promote muscular responses for strength and athletes are provided.

## 1. Introduction

Over the two past decades, there has been considerable interest in the effects of exercise-induced skeletal muscle growth, with previous research focusing on the influence of both acute [1,2,3,4,5,6] and chronic [7,8,9,10,11] biochemical events associated with resistance exercise. It is well-established that in order to maximize the hypertrophic response of skeletal muscle, it is necessary to optimize endocrine factors that promote protein synthesis and decrease protein degradation [12,13]. Recently, a greater research focus has been placed on specific nutritional supplementation strategies, such as liquid carbohydrate (CHO) [14,15,16], essential amino acid (EAA) [17,18,19], and mixed nutrient [20,21,22] ingestion in order to enhance acute biochemical responses to resistance exercise. However, as stated by Volek [23], little research has linked acute biochemical alterations following nutritive intervention to chronic musculoskeletal adaptations following resistance training [15,24,25].

This review will outline fundamental concepts related to nutritional physiology and provide a theoretical framework model [23] for answering some of the central questions surrounding resistance exercise and nutritive interventions, these being (i) nutrient quantity; (ii) nutrient quality; and (iii) nutrient timing. These are importance considerations given the recent work of King et al. [26], who reported the pre-, intra-, and post-exercise nutrition practices of 305 competitive powerlifters. Overall, 85.6% paid attention to pre-exercise fueling; 69.2% reported consuming food and/or calorie-targeted fluids during training (intra-exercise); while 80.0% followed post-exercise nutrition protocols. Therefore, this review aims to evaluate the efficacy of CHO, protein, and amino acid supplementation in enhancing muscular responses to resistance training in strength and power athletes and link such responses to the pathway of adaptation model [23]. This paper does not purport to be an exhaustive review of material published purely from a physiological perspective detailing resistance exercise and protein and amino acid metabolism. Readers are referred to other comprehensive reviews [27,28,29,30] for aspects of this topic that may not be addressed.

## 2. Pathway of Adaptation Model

**Figure 1:**
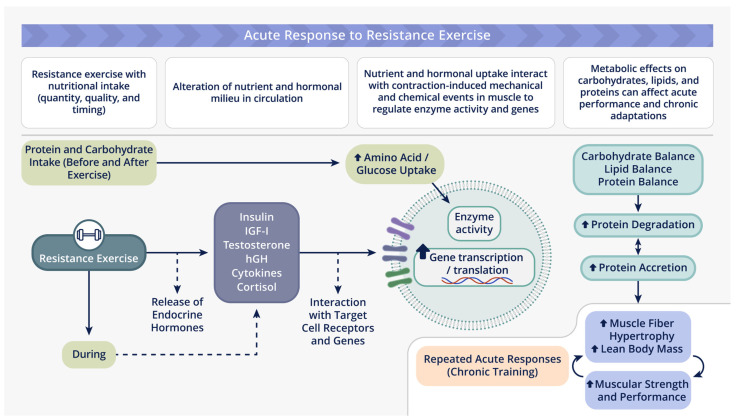
The pathway of adaptation model represents a theoretical chain of events demonstrating the influence of nutritional supplementation on acute resistance exercise and training. This results in chronic musculoskeletal adaptations that lead to skeletal muscle hypertrophy and increased strength expression. 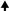, increase. Adapted from Volek [23].

The pathway of adaptation model, as proposed by Volek [23], provides a practical framework for the fundamental steps that mediate acute responses to resistance exercise associated with nutritional supplementation, and chronic musculoskeletal adaptations to training (Figure 1). This model is unique, as it outlines the chain of events deemed responsible for exercise-induced skeletal muscle growth and increased strength expression; these include (i) nutritive intervention (quality, quantity, and timing); (ii) contractile activity (acute resistance exercise); (iii) biochemical signals (hormonal events); and (iv) molecular programming (transcription/translation).

The basis for the pathway of adaptation model is attributed to the interaction of a number of physiological factors. Briefly, an acute bout of resistance exercise provides a potent stimulus that alters the secretion of both anabolic and catabolic hormones [31,32,33,34]; however, it is training that provides a chronic stimulus that promotes a number of cellular adaptations [35]. The degree to which cellular adaptations occur can be influenced not only by the resistance exercise prescription (i.e., acute program variables) [30], but also an individual’s nutrient status, which can be manipulated by various nutritional strategies. Past research has shown that nutritional supplementation can significantly influence the body’s hormonal response to resistance exercise, resulting in elevated insulin concentrations [31,34,36], while suppressing both testosterone [31,34,37] and cortisol [15,24,34], thereby altering the balance between hormone-mediated anabolic and catabolic activity.

The physiological relevance of the above findings is the contention that the combination of nutrient and hormone interaction may alter enzyme activity and genetic programming (i.e., the transcription and translation of proteins). Amino acids by themselves and in concert with hormones can modify the expression of target genes at the level of transcription, mRNA stability, and translation [38]. A review by Hamel et al. [39] proposed that amino acids and hormones (glucocorticoids) control the expression of the necessary components for the ubiquitin-proteasome pathway, thus playing a regulatory role in protein degradation. This provides a novel mechanism for the pathway of adaptation, one that is attributed to the suppression of protein degradation rather than enhancing protein synthesis [25]. Finally, if acute responses to resistance exercise are of sufficient magnitude and duration, chronic musculoskeletal adaptations will occur that lead to protein accretion and muscle fiber hypertrophy. This will result in increased strength expression in subsequent workouts, and directly influence the magnitude of the acute resistance exercise response. However, molecular and genetic predispositions may affect individual responses to both resistance exercise and nutritional interventions [40].

## 3. Nutritional Supplementation Strategies

Evidence from the scientific literature supports the notion that nutritional strategies enhance both the acute response to resistance exercise and the chronic adaptation to resistance training [15,17,24,25,31]. From a practical standpoint, Volek [23] outlines three important concepts that need to be considered when determining the effectiveness of nutritional strategies, these being (i) nutrient quantity (i.e., how much should be consumed?); (ii) nutrient quality (i.e., what kind of nutrients should be consumed?); and (iii) nutrient timing (i.e., when should nutrient intake occur?). These are important considerations when examining the literature, given that the interactive effects of different nutrients may influence the magnitude of the adaptive response to resistance training [25,36]. Other key considerations include the following: (i) the training status of the subjects; as majority of studies utilize untrained individuals, training status may influence the acute and chronic response to resistance exercise [7]; (ii) methodological differences in the measure of muscle protein metabolism (synthesis and degradation); for example, the clinical use of urinary 3-Methylhistidine (3-MHIS) as an indicator of muscle protein degradation has been met with criticism [41]; however, with careful dietary and exercise controls, qualitative inferences can be made concerning skeletal muscle metabolism using 3-MHIS [42,43,44], although stable isotope methods provide a more precise measure of acute changes in muscle protein turnover [45]; and (iii) dietary control measures; total kilocalorie intake and macronutrient composition should remain constant throughout the duration of the study [23]. As the primary focus of the current review is nutritional strategies that enhance skeletal muscle anabolism, the role of fat intake is not addressed. Readers are directed to recent reviews that address this issue regarding athletic performance [46,47]. Table 1 and Table 2 summarize research demonstrating the effects of nutritional supplementation following acute resistance exercise and chronic resistance training.

### 3.1. Carbohydrate Ingestion

CHO ingestion during and/or following resistance exercise has been examined based on two primary outcomes, these being (i) glycogen resynthesis and (ii) hormonal modification (for an in-depth review, see Conley and Stone [65] and Haff et al. [66]). First, the implementation of CHO supplementation may reduce muscle glycogen loss associated with an acute bout of resistance exercise. This may be of importance for athletes involved in multiple training bouts per day. Second, CHO ingestion may shift the exercise-induced hormonal milieu towards a profile more favorable for anabolism. Specifically, it is the response of insulin and cortisol that has received much attention, as these hormones have major regulatory roles in CHO metabolism [65].

#### 3.1.1. Glycogen Resynthesis

Several studies report a decrease in muscle glycogen following an acute bout of resistance exercise [67,68]. CHO ingestion influences glycogen resynthesis by increasing substrate availability to muscle, thereby enhancing uptake, and affecting gluco-regulatory hormones such as insulin, which may activate the steps involved in glycogen resynthesis. Although only a limited number of studies have examined the effects of CHO ingestion on muscle glycogen resynthesis following resistance exercise, the findings suggest that glycogen resynthesis is significantly greater with CHO consumption. Pascoe et al. investigated the influence of liquid CHO ingestion (1.5 g/kg) on glycogen resynthesis following a leg extension protocol (6 sets × 6 repetitions at 70% 1-RM) in six recreationally active college men [69]. The authors reported that muscle glycogen content depleted by 30% immediately following the exercise bout. However, CHO ingestion immediately and 60 min post-exercise resulted in significantly greater glycogen resynthesis during the initial 2 h recovery period compared to water only. Furthermore, after 6 h, muscle glycogen content was restored to 91% of pre-exercise levels following CHO ingestion and only 75% of pre-exercise levels following water only. This clearly indicates that CHO ingestion may be a powerful stimulator of glycogen sparing.

Roy and Tarnsopolsky [70] examined the effect of the energy intake of differing macronutrient compositions [CHO (1.0 g/kg), CHO/PRO/FAT (66% CHO, 23% PRO, 11% FAT) and PLA (placebo)] given immediately and 60 min post-exercise on glycogen resynthesis after a whole-body resistance exercise bout (9 exercises; 3 sets × 10 repetitions 80% 1-RM) in ten trained young men. Over three trials, resistance exercise resulted in an average decrease in muscle glycogen content of ~36%. Although the rate of glycogen resynthesis was significantly greater for the treatment conditions compared to PLA, interestingly, both the CHO and CHO/PRO/FAT conditions resulted in similar rates of muscle glycogen resynthesis. Therefore, the addition of PRO and FAT does not limit glycogen resynthesis. The authors speculated that these macronutrients may have contributed to gluconeogenic precursors, thereby increasing substrate availability.

Finally, Haff et al. reported that liquid CHO ingestion prior (1.0 g/kg) and during (0.5 g/kg) an acute bout of lower-body resistance exercise attenuated muscle glycogen loss in eight resistance-trained men [71]. The exercise bout consisted of 3 sets × 10 repetitions of back squats (65% 1-RM), speed squats (45% 1-RM), and one-leg squats (10% 1-RM). Ingestion of a placebo (PLA) beverage during the exercise bout resulted in a 26.7% decrease in muscle glycogen content. Conversely, a reduction of only 13.7% was observed after CHO ingestion. The authors suggest that elevated glucose levels associated with CHO ingestion limited muscle glycogen loss by either contributing to glycogen resynthesis or becoming a predominant fuel source as glycogen becomes depleted. Such a response may explain improved muscular performance during multiple-bout resistance exercise. Data presented by Lambert et al. [72] and Haff et al. [73] indicate that muscular performance measured as the number of sets and repetitions was greater following CHO ingestion than for PLA. Collectively, these data support the role for liquid CHO ingestion if glycogen sparing and/or resynthesis are required, such as when multiple sessions are performed on the same day.

#### 3.1.2. Hormonal Modification

Hormonal mechanisms that act within skeletal muscle are a part of an integrated system that mediates the adaptive response in the metabolic and cellular pathways. An extensive review by Kraemer and Ratamess [9] highlights that the hormonal response to resistance exercise takes place in a unique physiological environment. Interest lies in attempting to maximize the effectiveness of the hormonal response following an acute bout of resistance exercise, and when repeated, gaining the greatest potential for exercise-induced skeletal muscle growth. The addition of CHO supplementation to resistance exercise is suggested to enhance the anabolic environment, potentially increasing the hypertrophic adaptation of skeletal muscle [66]. However, the impact of hormones in mediating the effects of nutrition on acute and chronic responses to resistance exercise remains relatively unresolved. A first point of understanding how nutritional strategies affect exercise-induced hormones, such as insulin and cortisol, are the gluco-regulatory actions involved with CHO metabolism [65].

Several studies indicate that CHO ingestion modifies the acute hormonal response following a single bout of resistance exercise [13,31,34,74]. Most notably, insulin concentrations appear to reflect changes in glucose concentrations. Chandler et al. [37] demonstrated that CHO ingestion (1.5 g/kg) immediately before and 2 h after an acute bout of whole-body resistance exercise resulted in significantly higher insulin and glucose levels compared to PLA. Furthermore, the greater post-exercise insulin spike accompanied significantly higher growth hormone levels 6 h post-exercise. Conversely, testosterone concentrations decreased 30 min after supplementation and remained significantly lower throughout the 6 h post-exercise period. Similar results are presented by Kraemer et al. [32] using a combined CHO/PRO supplement (67% CHO, 33% PRO). Not only were insulin and growth hormone concentrations significantly higher after day 1 of three consecutive days of heavy resistance exercise following CHO/PRO supplementation, but testosterone also declined to below resting values. It is apparent that CHO and CHO/PRO supplementation result in significant increases in glucose, insulin, and growth hormone, while increased receptor uptake may account for the reduction in testosterone concentrations [75].

**Figure 2:**
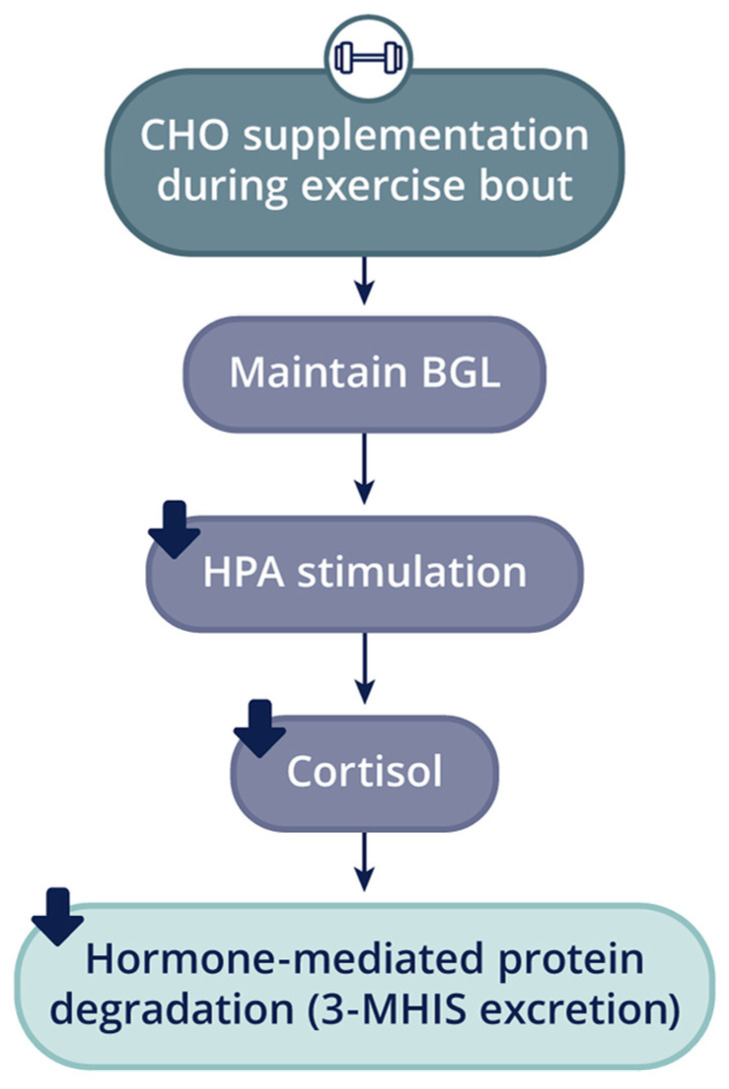
CHO delivery and stress response to resistance exercise. CHO ingestion during the exercise bout is proposed to sustain blood glucose levels and attenuate the stimulus for the adrenal cortex to secrete cortisol to catabolize cellular protein for gluconeogenic purposes. CHO, carbohydrate; BGL, blood glucose levels; HPA, hypothalamic–pituitary–adrenal axis; 3-MHIS, 3-methylhistidine. Adapted from Tarpenning and colleagues [15,53].

Research demonstrates that CHO ingestion alone, or combined with PRO, pre- and/or post-exercise improves net muscle balance, thus providing a basis for the hypothesis that nutritive intervention around the time of resistance exercise will positively impact the skeletal muscle hypertrophic adaptation to training [22,52,54]. Roy and colleagues found that CHO supplementation (1.0 g/kg) immediately and 1 h after unilateral knee extensor exercise (8 sets × 10 repetitions 85% 1-RM) significantly increased insulin and glucose levels compared to PLA [52]. Unique to this study were the findings of significant reductions in 3-MHIS and urea excretion, resulting in a more positive protein balance. The authors suggest that an increased insulin concentration was responsible for attenuating the post-exercise rise in protein degradation associated with an acute bout of resistance exercise. While Roy et al. [76,77] are of the opinion that the reduction in 3-MHIS excretion was the result of insulin’s ability to inhibit protein degradation, such an effect has not been demonstrated on myofibrillar proteins; therefore, it is possible that CHO-induced suppression of cortisol release contributed to the reduction in 3-MHIS excretion. Interestingly, cortisol values were not reported in the study by Roy et al. [52]. Alternatively, Tarpenning et al. [15] propose that the ‘anti-catabolic effect’ offered by liquid CHO ingestion is a result of CHO-induced blunting of the cortisol response (Figure 2). Previous work demonstrates that ingestion of a 6% CHO solution during an acute bout of resistance exercise attenuated exercise-induced cortisol release both during and after the exercise bout [15,42]. This response contrasts with significantly elevated cortisol levels occurring with resistance exercise alone of 99% and 105%, respectively.

However, not all studies agree. Koch et al. [78] had ten resistance-trained men perform a lower-body workout (squat exercise; 10 sets × 10 repetitions; progressive 40% to 85% of 1-RM) while ingesting a 20% CHO solution (maltodextrin and dextrose) or a PLA beverage pre- and post-exercise. The authors reported significant increases in the immediate post-exercise cortisol response (29% for CHO; 42% for PLA), with no differences between the groups. Using a CHO load of 1 g/kg, similar findings are presented by Thyfault et al. and Williams et al., as CHO ingestion 10 min pre- and post-exercise and immediately post-exercise did not result in an attenuation of the exercise-induced elevation in cortisol following resistance exercise [16,34]. One must consider the methodological differences when comparing studies, such as exercise protocol (intensity and duration) and CHO concentration (which affects gastric emptying time). Collectively, such differences may elicit differences in cortisol response between studies.

Due to multiple factors inherent in different experimental designs (i.e., resistance exercise program design; time course of CHO administration; amount of CHO), it is difficult to resolve the discrepancies between the opposing results of the previously cited studies. However, some general conclusions may be drawn. The gluco-regulatory action of cortisol is maximally stimulated by high-intensity resistance exercise; therefore, limiting CHO ingestion to pre- and/or post-exercise may be of little value in an attempt to suppress this hormone action. Further, different types of CHO have different digestive properties. Jeukendrup and Jentjens [79] suggest that differences in osmolality and structure may have effects on digestion and absorption and the availability of glucose for oxidation in the muscle.

The CHO concentration appears to be an important regulator of the rate of gastric emptying. Murray et al. [80] reported that the gastric emptying rate of a 20% CHO beverage was significantly slower than a 6% CHO solution, and the effect became increasingly apparent with time. Approximately 20 min after ingestion, only 45% of the original volume of 20% CHO had been emptied from the stomach, whereas 75% of the 6% CHO had emptied. Additional research examining the gastric emptying rates following ingestion of a 4%, 6%, and 8% CHO solution during exercise indicated that although the osmolalities of the 6% and 8% solutions were similar, ingestion of the higher CHO concentration (8%) resulted in significantly slower gastric emptying rates [81]. The authors concluded that CHO concentration, rather than osmolality, is the primary factor influencing gastric emptying rates. Therefore, it appears that increasing the CHO content will reduce the emptying rate, decreasing the rate of fluid delivery. Collectively, these findings may provide an explanation for the inability of higher CHO concentrations to provide a suppressive effect on exercise-induced cortisol release, as reported in the above investigations.

The physiological implication of cortisol’s action in hormone-mediated protein degradation may be inferred from the recent work of Bird, Tarpenning, and Marino [42]. The authors reported that following an acute bout of resistance exercise in untrained young men, total tissue exposure to cortisol, as determined by area under the curve (AUC) values, was significantly greater following consumption of a PLA beverage, compared to CHO and/or EAA ingestion, and this corresponded with a substantial increase in 3-MHIS excretion 48 h following the exercise bout. Further, a positive linear association (r = 0.75, *p* < 0.05) was observed between the change in myofibrillar protein degradation and cortisol AUC values for the PLA group. However, no significant associations were reported between exercise-induced cortisol release and 3-MHIS excretion following either liquid CHO or EAA ingestion. Conversely, a negative linear association (r = −0.77, *p* < 0.05) was observed following CHO+EAA ingestion [42].

This raises the question of whether transient improvements in protein turnover following CHO ingestion result in greater gains in skeletal muscle growth. However, few studies have examined the effect of acute CHO ingestion on chronic muscular adaptations following resistance training. Tarpenning et al. [15] showed that ingestion of a 6% CHO solution not only blunted the exercise-induced cortisol response following resistance exercise, but this altered response was associated with significantly greater gains in both type I (r = −0.86, *p* < 0.01) and type II (r = −0.72, *p* < 0.05) fiber cross-sectional area (fCSA) compared to resistance training without nutritive intervention. Additional research confirmed the original findings, with CHO consumption resulting in a significant increase in fCSA for type I fCSA (18.4%) and type IIa (16.3%), relative to a PLA-only control (7.1 and 8.2%, respectively), whereas, a trend was displayed for type IIb (13.6 vs. 6.8%, respectively) [25]. Interestingly, the relative increase in hypertrophy between fiber types appears to be mediated, at least to some extent, by the independent effects of CHO and EAA ingestion, as EAA ingestion resulted in similar increases in fCSA to that of CHO. In contrast, Andersen et al. reported that 14 weeks of lower-body resistance exercise combined with CHO ingestion (25 g maltodextrin) did not result in long-term skeletal muscle anabolism [24]. Therefore, further research is required to elucidate these findings and unify the mechanisms responsible for muscular adaptations following CHO ingestion.

In summary, a review of the findings suggests that liquid CHO ingestion at lower CHO concentrations (6–8%) results in modification of the acute hormonal response, and when repeated, alteration of the gluco-regulatory hormones, insulin and cortisol. Such a response may produce reductions in protein degradation, thereby increasing protein anabolism and skeletal muscle growth. Furthermore, liquid CHO ingestion offers a muscle glycogen sparring effect, thereby enhancing muscular performance associated with resistance exercise.

### 3.2. Protein and Amino Acid Ingestion

Accretion of muscle protein as a result of resistance training occurs from a positive net protein balance (protein synthesis minus protein breakdown). Although at any given time, the net protein balance could be either positive or negative, the overall summation of the protein balance over time must be positive. Amino acid availability plays an important role in controlling muscle protein kinetics, with intact protein (PRO) ingestion and amino acid administration, either orally or intravenously, resulting in an enhanced anabolic environment, protein synthesis, amino acid transport, and a reduction in protein degradation [17,48,50,55,82,83]. However, following an acute bout of resistance exercise, rates of protein synthesis and degradation are acutely elevated, but the net muscle protein balance is negative (i.e., protein degradation exceeds synthesis) if subjects remain in a fasted state [84,85]. Without amino acid ingestion, protein synthesis may be limited due to decreased amino acid availability [86]. An attractive hypothesis, as proposed by Bohe et al. [87] is that the deposition of amino acids into tissue by protein synthesis and their release by protein degradation is mediated by the concentrations of amino acids, particularly EAA. That is to say, the EAA signal to the protein synthetic machinery occurs via receptors which are extracellular, not intracellular, and this may be potentiated by small doses of EAA. Therefore, an increase in extracellular amino acid availability may provide a potent signal for augmenting the hypertrophic response of skeletal muscle following resistance exercise.

#### 3.2.1. Protein Synthesis

**Figure 3:**
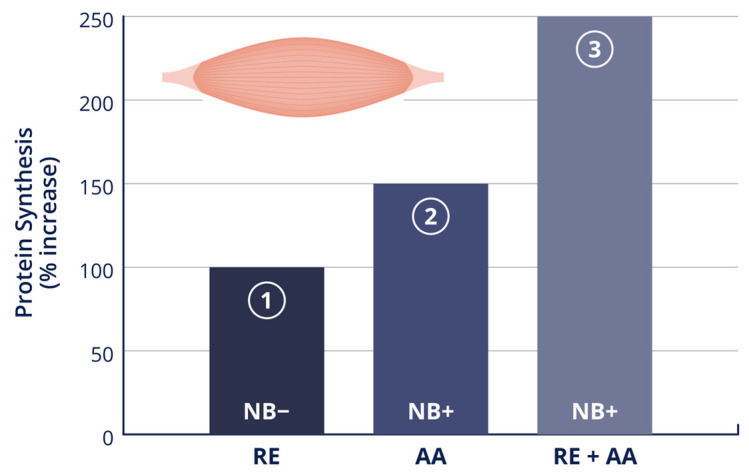
Resistance exercise and amino acid ingestion has an additive effect on protein synthesis. (1) Following an acute bout of resistance exercise, protein synthesis increased by ~100%, whereas protein breakdown increased by ~50% (net protein balance: negative); (2) at rest with increased amino acid availability, protein synthesis increases by ~150% (net protein balance: positive); and (3) after resistance exercise with increased amino acid availability, protein synthesis increases by >200% (net protein balance: positive). RE, resistance exercise; AA, amino acid; NB, net balance. Adapted from Biolo et al. [48,84].

Amino acid ingestion before and/or after resistance exercise has an additive effect on protein synthesis (Figure 3). Tipton et al. [17] had subjects consume 40 g of either mixed or EAA in one liter of water during ~4.5 h of recovery from an acute bout of lower-body resistance exercise. The net muscle balance was negative during the PLA condition; however, this changed to positive following amino acid ingestion. The authors concluded that post-exercise amino acid ingestion results in a positive muscle protein balance; however, it does not appear necessary to include nonessential amino acids, as the net balance was similar for mixed and EAA. Using an EAA composition designed to increase muscle amino acids levels in proportion to their requirements for protein synthesis, Rasmussen et al. [19] reported that, when compared to PLA, 6 g of EAA combined with 35 g of sucrose promoted a transient net increase in muscle protein balance during the hour immediately following ingestion [22]. This may indicate that a relatively small dose of EAA can markedly stimulate muscle protein synthesis; however, the independent effect of EAA and CHO ingestion was not assessed in this investigation.

To address this issue, Borsheim et al. [18] determined the independent effect of ingesting 6 g of EAA administered at 1 and 2 h post-exercise on net muscle protein synthesis. The authors found that ingesting 6 g of EAA during recovery produced a transient net increase in muscle protein balance that was comparable to that observed by Rasmussen et al. [22]. It is noteworthy that the overall magnitudes of change in muscle protein balance reported above were even higher than the values reported by Tipton et al., who provided 40 g of EAA [17]. However, a direct comparison between studies is limited by methodological considerations. For example, some researchers [18,22] have calculated net muscle protein balance in hourly blocks following ingestion, whereas others have averaged the muscle response over a 4 h period [17], and this may have underestimated the acute increase that occurred.

More recently, it has been suggested that the ability of a food source to stimulate muscle protein synthesis is dependent on its protein digestibility, amino acid kinetics, and the amino acid composition [88]. Studies consistently show that post-exercise muscle protein synthesis is significantly elevated after the ingestion of rapidly digesting amino acids and especially EAAs [89,90]. Of all available EAAs, the branched chain amino acid (BCAA) leucine has been shown to upregulate the mechanistic target of rapamycin complex 1 (mTORC1), which is a signaling cascade that increases muscle protein synthesis [91]. Due to these findings, the concept of the “leucine trigger” has been hypothesized, specifically that larger increases in blood leucine concentrations lead to greater stimulation of muscle protein synthesis [92]. Zaromskyte and colleagues [93] conducted a systematic review of the available literature in attempt to validate this theory. In their review, they identified a total of 29 studies that examined muscle protein synthesis rates in response to leucine ingestion. Of the 29 total studies, 16 show sufficient evidence to support the hypothesis, though the results appeared to be greater in older adults. They posited that the importance of consuming leucine was more relevant when ingesting isolated protein sources, rather than protein-rich whole foods.

Elliot et al. [94] examined the response of the net muscle protein balance following resistance exercise and ingestion of PRO in the context of a food. Subjects were randomly assigned to one of three groups receiving a milk drink (237 g fat-free milk [FM], 237 g whole milk [WM] or 393 g isocaloric fat-free milk [IM]) 1 h following a leg resistance exercise routine (knee extensor exercise; 10 sets × 8 repetitions; 80% 1-RM). Arterial concentrations of phenylalanine and threonine increased in response to milk ingestion, with the net balance for both amino acids changing from negative to positive across groups. Net uptake for threonine was 2.8-fold greater (*p* < 0.05) for WM and 2.5-fold greater for IM, compared to FM. The mean uptake of phenylalanine was 80 and 85% greater for WM and IM, respectively, than for FM. Threonine uptake relative to the amount ingested was 312% greater (*p* < 0.05) for WM and 91% for IM, than FM. Mean phenylalanine uptake/ingested also was greatest for WM. The authors concluded that milk ingestion following an acute bout of resistance exercise results in the uptake of phenylalanine and threonine, which is representative of net muscle protein synthesis. Therefore, ingestion of whole milk may provide an alternative to supplements for strength and power athletes wishing to enhance recovery from resistance exercise.

The physiological role of insulin is an important consideration when examining the influence of amino acid ingestion on the hypertrophic response of skeletal muscle. The mechanisms implicated in insulin’s anabolic action in the stimulation of muscle protein synthesis include increases in the activation of enzymes, amino acid availability, and the translation of mRNA and gene transcription [95]. Although insulin is required for muscle protein synthesis to proceed, it appears that insulin is not the primary regulator [22]. In the absence of an increase in amino acid concentration, an increase in insulin has only a modest effect on muscle protein synthesis [84]. The lack of an effect of insulin on protein synthesis following resistance exercise is suggested to reflect a deficiency in amino acid availability, rather than a deficit in the direct stimulatory effect of insulin on protein synthesis [96]. Therefore, supplying small doses of EAA during or near the time of the exercise bout should allow the expression of the stimulatory effect of insulin. Accordingly, Tipton et al. [17] have shown that rates of protein synthesis and net protein balance are greater when amino acid availability is increased following an acute bout of resistance exercise than when subjects are fasted. It is likely that elevations in amino acid concentrations and increased insulin explain a potent effect in promoting protein synthesis and net protein deposition in skeletal muscle [97].

#### 3.2.2. Protein Type

When amino acids or PRO are ingested, the extent of increase in amino acid concentrations depends not only on the relative contribution in the blood entering the muscle, but also the individual transport kinetics [95]. The general concept underlying this approach, as suggested by Mahe et al. [98], outlines differences in the digestive properties of PRO. For example, casein clots in the stomach, which delays gastric emptying, resulting in a sustained ‘slow’ release of amino acids, whereas whey PRO is a soluble ‘fast-acting’ PRO [98]. Data presented by Boirie et al. [99] indicate that amino acids released from casein appear in the blood more slowly and peak at a lesser magnitude; however, the response lasts longer than for whey PRO. The authors concluded that the speed of amino acid absorption has a major impact on protein turnover. In resting subjects, the slowly absorbed casein promotes protein accretion by the inhibition of protein degradation; by contrast, a fast-acting PRO (whey) stimulates protein synthesis. Additional data revealed that the pattern of appearance is the critical factor, rather than the amino acid composition of the PRO [100,101].

With differences in the digestive properties of casein and whey PRO established [98,100,101], Tipton et al. [55] examined whether such differences would impact the net muscle protein balance following an acute bout of lower-body resistance exercise. Subjects were randomly assigned to one of three groups and received 20 g casein, 20 g whey PRO, or a PLA beverage 60 min post-exercise. Although the insulin response, as determined by AUC values, was twice as high following whey ingestion compared to casein, both groups improved their net muscle protein balance to positive, indicating that any difference in muscle responsiveness to the two PROs was not large. This is an interesting finding, considering the pattern of amino acid appearance in the blood was markedly different following casein and whey ingestion. It is possible that the stimulation of muscle protein metabolism by resistance exercise may alter the response to casein and whey PRO ingestion. The authors concluded that post-exercise consumption of whole PRO may be an effective strategy for promoting maximal gains in muscle mass and strength expression following resistance training.

The 2023 Position Statement by the International Society of Sport Nutrition [102] highlights that EAA ingestion in free form has been shown to be effective in stimulating MPS in doses as small as 1.5 g. Since it is readily absorbable, free-form EAA ingestion results in a more rapid increase in blood EAA concentrations and a subsequently greater peak in intramuscular EAA concentrations compared to whole protein sources [103,104]. Given the decreased rate of gastric emptying and subsequent digestion and absorption of whole protein sources, free EAA ingestion may be the preferred method of increasing blood EAA levels pre-workout.

Recent research examining the effects of different combinations of leucine, whey, and casein suggests an ergogenic effect that promotes increases in fat-free mass and strength expression beyond that achieved with resistance training and CHO ingestion [43,64,105]. Coburn et al. [105] compared the magnitude of change in strength response to 8 weeks of unilateral knee extensor resistance exercise (periodized; 3–5 sets × 6 repetitions at 80% of 1-RM; 3 d/wk) in de-trained (no resistance exercise in past 90 days) young men. Subjects consumed either 20 g whey PRO plus 6.2 g of leucine (SUPP), 26.6 g matodextrin (PLA), or nothing (CON) 30 min prior to and immediately-post exercise. Interestingly, significant increases in 1-RM strength were reported for both limbs in SUPP (30.4 and 14.5%, respectively), but only the trained limb (22.4%) in PLA. Furthermore, the increase in strength for the trained limb of SUPP was significantly greater than that for the trained limb of PLA. The authors suggest that the supplement may have accentuated the cross-training effect of the untrained limb.

Kerksick et al. [64] investigated whether two different forms of PRO supplementation (40 g of whey PRO and 8 g of casein PRO [WC], or 40 g of whey PRO plus 5 g of L-glutamine plus 3 g of BCAA [WBG]) would enhance muscular adaptations (strength and body composition) following 10 weeks of resistance training (upper/lower split; periodized; 3 sets × 6–10 RM; 4 d/wk) in comparison to an isoenergetic amount of CHO (48 g of CHO [PLA]). The WC group was the only group that showed significant increases in lean mass (WC = 1.9 kg; PLA: no change; WBG = −0.1 kg, *p* < 0.05) and fat-free mass (WC = 1.8 kg; PLA = 0.1 kg; WBG = −0.1 kg, *p* < 0.05), with a trend (*p* = 0.054) toward a greater increase in body mass (WC = 3.0 kg; PLA: 0.2 kg; WBG = no change). Significant increases in 1 RM strength for bench press and leg press were observed across groups after 10 weeks. The authors concluded that supplementing the diet with a whey/casein mix while resistance training improves muscular adaptations to a greater extent in comparison to ingestion of CHO alone.

In summary, these data clearly illustrate the anabolic response of skeletal muscle protein to EAA ingestion and provide further evidence that different types of PRO have varying effects on muscular adaptations to resistance training. The effect of EAA is enhanced by an elevated insulin concentration after CHO ingestion [19,22]. As such, there appears to be a requirement for a minimal concentration of insulin to stimulate protein synthesis in response to resistance exercise and amino acid ingestion, and the effects are integrated at the level of one central regulatory protein, the mammalian target of rapamycin (mTOR) [106]. Collectively, these data indicate that the potential ergogenic value of PRO supplementation during the early phases of training may vary depending on the specific amino acid composition of the supplement. Therefore, nutritive interventions centered on mixed PRO intake (whey, casein, and EAA ingestion) around the time of exercise are an integral component necessary to potentiate the effects of resistance exercise on skeletal muscle growth.

### 3.3. Combined Ingestion of Carbohydrate, Protein, and/or Amino Acids

Optimizing the quality of nutrient intake (i.e., combining CHO, PRO, and/or EAA) may offer a sustained response over the postprandial period. Therefore, the potential role of mixed nutritive ingestion to increase both extracellular amino acid availability and insulin release, as well as suppressing exercise-induced cortisol secretion, should provide a potent stimulator for positive protein balance [15,22,31,54]. Such a strategy remains a realistic potential mechanism aimed at conservation of myofibrillar protein, thereby enhancing skeletal muscle growth by suppressing myofibrillar protein degradation [31]. This provides the basis for the hypothesis that mixed nutritive ingestion pre, during, and/or post-exercise will provide transient improvements in protein balance, and when repeated, will result in greater gains in muscle mass and strength expression following chronic resistance training.

Examining the influence of nutrient timing on protein synthesis, Tipton et al. [54] determined whether an oral amino acid-carbohydrate (EAC) supplement would be a more effective stimulator of muscle protein anabolism if given immediately before or immediately after an acute bout of lower-body resistance exercise. Ingestion of EAC (6 g EAA plus 35 g sucrose) resulted in a change in muscle protein balance from negative (net release) to positive (net uptake) in both trials. More importantly, the total response to pre-exercise EAC ingestion was significantly greater than the response when EAC was consumed post-exercise. The authors postulate that the combination of increased amino acid availability at a time when blood flow is increased offers the maximum stimulatory effect required for enhanced protein synthesis.

This contention is supported, in part, by Borsheim, Aarsland, and Wolfe [49], who investigated the time course of the anabolic response to a single bout of lower-body resistance exercise following ingestion of a whey PRO, amino acids (AA), and CHO (PAAC) beverage or CHO solution (100 g maltodextrin) 60 min post-exercise. The net phenylalanine balance increased rapidly from negative to positive following PAAC ingestion. An initial peak in the phenylalanine net balance occurred only 20 min after ingestion, which was followed by a decline. Interestingly, after this first rapid decrease, the net balance increased to a new peak 90 min after the drink. Conversely, no change was observed following CHO consumption. Therefore, the net protein balance response to PAAC appeared to consist of at least two phases: an initial stimulatory response, due to the AA in the mixture, and a later response, possibly due to the anabolic effect of insulin. The authors concluded that ingestion of a whey PRO, AA, and CHO mixture stimulated net muscle protein synthesis to a greater extent than CHO alone. Further, the addition of whey PRO to AA+CHO results in a lasting response greater than the first hour after ingestion.

In agreement, Koopman et al. [51] reported that CHO, PRO, and free leucine (CHO+PRO+Leu) ingestion immediately following 16 sets of lower-body resistance exercise improved the whole-body protein balance during recovery compared to CHO or CHO+PRO consumption. Furthermore, CHO+PRO+Leu ingestion augmented protein synthesis to a greater extent than CHO ingestion. Not only was the leucine concentration significantly higher during the CHO+PRO+Leu trial, but the observed insulin response was also correlated (r = 0.77; *p* < 0.001) with the leucine concentration. Most importantly, this study offers a mechanistic model for the basis of protein synthesis following acute resistance exercise. The authors outline that the stimulatory effects of leucine and insulin on protein synthesis involves signaling through mTOR. Therefore, maximal rates of protein synthesis require the anabolic signals of leucine and insulin and activation of mTOR, and these signals were substantially elevated during the CHO+PRO+Leu trial [51,97].

**Figure 4:**
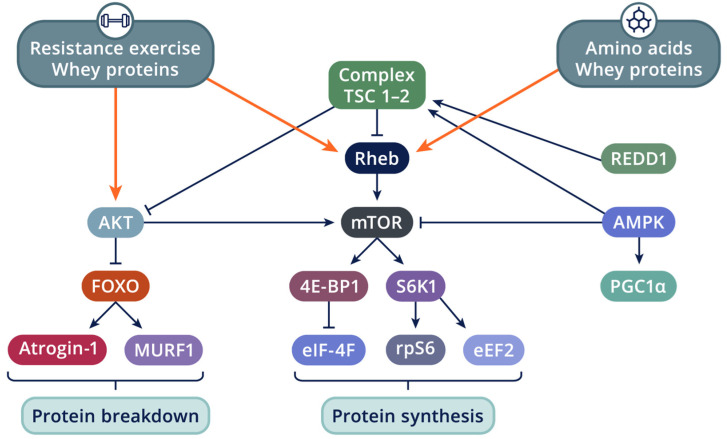
Molecular pathways that lead to protein synthesis and breakdown. (4E-BP1: eukaryotic translation initiation factor 4E binding protein 1; Akt, protein kinase B; AMPK, adenosine monophosphate-activated protein kinase; eEF2, eukaryotic elongation factor-2; eIF-4F, eukaryotic translation initiation factor-4F; FOXO, forkhead box O; mTOR, mammalian target of rapamycin; MURF1, muscle RING-finger protein-1; PGC1α, peroxisome proliferator-activated receptor gamma coactivator 1-alpha; REDD1, regulated in development and DNA damage response 1; Rheb, Ras homolog enriched in brain; rpS6, ribosomal protein S6; S6K1, p70 ribosomal protein S6 kinase; TSC1-2, tuberous sclerosis 1–2).

However, the central question considered here is the importance of acute changes in muscle protein net balance that occur with nutritive intervention and whether such changes act as a regulatory process in skeletal muscle growth [107]. To address this issue, Bird, Tarpenning, and Marino [25] examined chronic alteration of the acute hormonal response associated with liquid CHO and/or EAA ingestion on hormonal and muscular adaptations following 12 weeks of resistance training. Thirty-two untrained young men randomly assigned to one of four groups (6% CHO solution, 6 g EAA mixture, combined CHO+EAA supplement, or PLA) performed a complete-body resistance exercise bout (8 exercises; 3 sets × 10 repetitions 75% 1-RM) twice per week, during which they consumed ~675 mL during the exercise bout. Diets were controlled, with energy intake set at the RDI of 9.8–13.7 MJ·d–1 based on height and body mass. A hypertrophic effect was observed for all three fiber types across groups (Figure 4); however, the CHO+EAA group demonstrated the greatest relative increase in fCSA for type I (23.4%), type IIa (27.1%), and type IIb (20.4%).

Although the net muscle protein balance was not determined in this study, the significant hypertrophy demonstrated by the CHO+EAA group is suggested to be the product of an “anti-catabolic effect” [25]. The authors propose that such a response is mediated by the interaction of two separate mechanisms. The first involves a transient decrease in hormone-mediated protein degradation, via suppressing exercise-induced cortisol release. The second is a transient increase in protein synthesis, via increased extracellular amino acid availability. This contention is supported, in part, by Tipton et al., who reported that the ingestion of 6 g EAA + 35 g sucrose, either pre- or post-exercise, not only inhibits protein breakdown (as assessed by phenylalanine Ra) but also stimulates protein synthesis [54]. The stimulation of protein synthesis by EAA, in addition to the inhibition of the normal post-exercise rise in protein degradation, is likely to be accounted for by the synergistic effect of the drink.

Several studies have shown that resistance training combined with nutritive intervention results in significant changes in body composition [54,57,61,108,109]. Gater and colleagues [108] reported that 10 weeks of resistance training combined with a mixed macronutrient beverage resulted in significant increases in both body mass [BM] (3.8 kg) and fat free mass [FFM] (3.6 kg) compared to PLA (1.6 and 2.1 kg, respectively). Following 8 weeks of resistance training, Rozenek et al. [109] observed similar increases in both BM (3.1 and 3.1 kg, respectively) and FFM (2.9 and 3.4 kg, respectively) for subjects consuming either a liquid CHO+PRO supplement or an isocaloric CHO beverage, which were significantly greater compared to a resistance-training-only control group. In agreement, Chromiak et al. [57] reported that ingesting a recovery drink containing whey PRO, CHO, amino acids, and creatine post-exercise did not result in greater improvements in any parameters of body composition compared with a CHO-only control. However, there was a trend (*p* = 0.07) toward a greater increase in FFM for the supplement group (3.4 kg) compared with the CHO control (1.5 kg). Furthermore, there were no improvements in the performance variables (muscle strength, muscle endurance, and anaerobic performance) when compared with the CHO-only control.

Finally, examining the independent and combined effects of liquid CHO and/or EAA ingestion, Bird, Tarpenning, and Marino [25] reported similar changes in body composition following 12 weeks of resistance training in untrained young men. CHO ingestion resulted in increases in BM and FFM of 1.8 and 2.9 kg, respectively, and this was matched by an increase of 1.9 and 3.0 kg, respectively, for the EAA group. It is difficult to ascertain whether the benefits are due to additional energy intake or the specific macronutrient mix of the supplements; however, the authors suggest that the independent effects of CHO and EAA ingestion may stimulate similar pathways [25,61]. Furthermore, the effects appear to be additive when treatments are combined, with significantly greater gains in BM (2.8 kg) and FFM (4.1 kg) displayed by the CHO+EAA group relative to PLA (0.4 and 1.8 kg, respectively).

In summary, the combinations of nutritive interventions described above clearly demonstrate the synergistic effect of mixed nutrient ingestion in promoting skeletal muscle anabolism. Thus, a combination of EAA and CHO to increase amino acid availability and insulin release, as well as suppressing exercise-induced cortisol secretion, may provide an optimal nutritional composition for the stimulation of muscle growth [15,22,31,54]. Therefore, mixed nutrient ingestion (pre-exercise, during the exercise bout, and/or post-exercise) is an integral component that can favorably influence the key steps in the pathway of adaptation model [23]. Further research is required to examine the influence of the addition of other amino acid derivatives, such as creatine and β-Hydroxy-β-methylbutyrate (HMB), as their inclusion may potentiate muscular adaptations to resistance training [110].

## 4. Resistance Exercise, Nutrition Supplementation Strategies, and Molecular Responses

An interaction between resistance exercise and nutrition is necessary to reach an optimal balance between anabolic and catabolic processes [42,54,111,112]. At a molecular level, mechanotransduction, combined with nutritional strategies, alters the signaling cascades responsible for protein breakdown and synthesis [92,113,114,115]. In this section, recent findings in this field of research will be explored.

### 4.1. Resistance Exercise and Molecular Responses

Resistance training is known to influence hormonal and metabolism responses during exercise and the post-exercise recovery period. The resultant impact on the signaling cascade for protein synthesis (Figure 4) starts with protein kinase B (or Akt) and via the stimulation of guanosine triphosphate (GTP) binding to the small G protein Ras homolog enriched in brain (Rheb), the mammalian target of rapamycin (mTOR) will be activated [116,117]. The mTOR signal transduction pathway plays a key regulatory role in the mRNA translation initiation of proteins [118]. The activity of this signaling cascade is largely determined by the phosphorylation of mTOR and the subsequent activation of p70 ribosomal protein S6 kinase (p70S6K, also noted in the literature as S6K1) and eukaryotic initiation factor eIF4E-binding protein-1 (4E-BP1) [119]. Muscle contractions also activate mTOR via phosphatidic acid [115] and the downregulation of regulated in development and DNA damage response 1 (REDD1). The importance of REDD1 is underscored by the ability of REDD1 to dominantly suppress mTOR activity even in the presence of the strong growth signal elicited by a myristylated form of Akt [120]. Furthermore, resistance training increases muscle size and strength gains via the activation of satellite cells or myogenic precursor cells (PAX7, NCAM, FAI1) and myogenic regulatory factors (MYF5, MYOD, MYOGENIN), responsible for muscle growth and repair [121].

A key regulator of energy metabolism is peroxisome proliferator-activated receptor gamma coactivator 1-alpha (PGC-1α). Previous research demonstrated that resting levels of peroxisome proliferator-activated receptor-γ coactivator (PGC)-1α4 mRNA expression were increased in healthy adults after resistance training [122,123]. Resistance exercise also alters the activity of mitogen-activated protein kinases (MAPK) involved in regulating architectural remodeling processes in muscle tissue. The authors of [124,125] observed that MAPK activation (p38MAPK, ERK1/2, p90RSK) was specific to eccentric-only resistance training. They also showed that AKT-mTOR or inflammatory signaling was not affected 30 min after exercise with both eccentric and concentric-only resistance training.

Additionally, resistance training influences the inflammatory pathway tumor necrosis factor alpha (TNFα)—Muscle RING-finger protein-1 (MuRF-1)—muscle atrophy F-box (MAFbx) [125]. The two latter molecules are muscle-specific E3 ubiquitin ligases, and together with FBXO32 (also known as atrogin-1), they are principal mediators of ubiquitin proteasome pathway (UPP) protein degradation [126]. Several studies showed that single bouts of traditional resistance exercise increase MuRF-1 mRNA 1–4 h post-exercise [127,128]. Stefanetti et al. [129] also observed that resistance training upregulated atrogin-1, FBXO40, FOXO1, and FOXO3 mRNA. In the trained state, however, single-bout resistance exercise decreased FBXO40 mRNA and protein. Additionally, the activation of Akt downregulates the expression of MuRF-1 and MAFbx [130].

In general, resistance exercise triggers protein synthesis by activating myogenic precursor cells and myogenic regulatory factors, as well as different signaling pathways, such as Akt-mTOR, PGC-1α, and MAPK. Resistance exercise also activates muscle catabolism through the inflammatory pathway TNFα—MuRF-1—MAFbx.

### 4.2. Interactions between Resistance Exercise, Carbohydrate, Protein, and Amino Acid Ingestion on Molecular Signaling

Proteins consumed before, during, and after resistance exercise increase muscle protein synthesis rates [35,89]. It has been demonstrated that amino acids activate Rheb, a low-molecular-weight GTPase located immediately upstream of mTOR (Figure 4), and human vacuolar protein sorting-34 (hVps34), both involved in mechanisms by which amino acids augment protein synthesis through mTOR signaling [131,132]. Through mTOR activation, several downstream proteins will be activated, including p70S6K and eukaryotic elongation factor-2 (eEF2). On the other hand, 4E-BP1 will be inactivated. This pathway triggers the activation of mRNA translational initiation and elongation [133,134,135].

Essential amino acids activate anabolic signaling. Leucine, one of the essential amino acids, is a branched chain amino acid (BCAA) and unique amongst the amino acids in its capacity to stimulate both mTOR and 4E-BP1 phosphorylation and to enhance p70S6K signaling [136]. Areta et al. [137] determined the effect of increasing concentrations of leucine on the early mTOR-mediated intracellular signaling response and rates of protein synthesis, as well as chronic changes in cell growth/diameter, in C2C12 muscle cells. The findings of the latter study indicate that even at low leucine concentrations, the phosphorylation of proteins regulating translation initiation signaling is enhanced. Furthermore, the phosphorylation of p70S6K follows a leucine dose–response relationship, but this was not reflected by the acute protein synthetic response. It also seemed that leucine concentrations of at least 5 mM are necessary to enhance cell growth.

Some research focused on the effect of BCAA and resistance training on molecular adaptations. Karlsson et al. [138] investigated the effect of resistance exercise, alone or in combination with the oral administration of BCAA, on the phosphorylation of p70S6K. Resistance exercise led to a robust increase in p70S6K phosphorylation, which persisted 1 and 2 h after exercise. Ingestion of BCAA increased plasma concentrations of the essential amino acids, isoleucine, leucine, and valine, during exercise and throughout recovery after exercise (2 h post-exercise), whereas no change was noted after the placebo trial. Borgenvik et al. [127] determined that BCAA ingestion increases the phosphorylation of p70S6K in the early post-exercise recovery period compared to the placebo condition. However, the authors also observed reduced muscle levels of phenylalanine and tyrosine during the post-resistance exercise, which attenuated muscle levels of essential amino acids. Apró and Blomstrand [139] did not observe a beneficial effect on the phosphorylation of p70S6K with resistance exercise alone, but BCAA intake increased phosphorylation in both legs following exercise. They also found that the phosphorylation of Akt was unaltered by either resistance exercise and/or BCAA supplementation, whereas mTOR phosphorylation was enhanced.

The effect of resistance exercise and BCAA alone or in combination on protein breakdown signaling pathways, as determined by Borgenvik et al. [127], indicates that after resistance exercise an increase in mRNA and the protein expression of MuRF-1 is observed in contrast to MAFbx. The latter observation likely reflects an increased degradation of contractile proteins. Supplementation with BCAA reduced the expression of MAFbx mRNA in resting and exercising muscle and prevented the increase in MuRF-1. The decreased level of MAFbx mRNA following BCAA supplementation suggests the reduced breakdown of regulatory proteins involved in hypertrophic signaling mediated by mTOR.

One of the strategies to faster increase blood BCAA concentrations is with the ingestion of whey proteins. Lam et al. [140] conducted a systematic review and meta-analysis to assess the efficacy of whey protein supplementation on blood BCAA levels. A total of 15 studies were identified, and the results of the meta-analysis showed a 458 nmol/L increase in blood BCAA levels after whey protein supplementation, compared to the control group. Whey protein significantly increases the phosphorylation of mTOR compared to a placebo drink in both younger and older subjects [141]. Additionally, the evidence showed that whey protein administration resulted in a higher phosphorylation of p70S6K, eIF4G, and 4EBP1 for older subjects, and rpS6, eIF4G, and 4EBP1 tended to increase in the younger subjects. Kakigi and colleagues [142] add to the literature that the ingesting of whey protein after concentric knee extension exercise in untrained men increases the phosphorylation of Akt and mTOR in a dose-dependent manner, in contrast to no protein intake. Additionally, whey protein intake after resistance training increases 4E-BP1 and further increases p70S6K phosphorylation, when 20 g of protein is administered compared to 10 g of protein.

The impact of protein ingestion and/or carbohydrate intake before and after exercise on ribosomal p70S6K phosphorylation status in human skeletal muscle tissue was investigated by Koopman et al. [143]. During recovery, p70S6K phosphorylation remained higher in carbohydrates and proteins than in the carbohydrates-only condition. However, Ferreira et al. [144] found that peri-exercise co-ingestion of carbohydrates and BCAA did not augment resistance exercise-induced increases in skeletal muscle signaling markers indicative of muscle protein synthesis, when compared with carbohydrates alone in non-resistance-trained males. The ingestion of carbohydrates, or carbohydrates in combination with BCAA, augments resistance exercise-induced insulin receptor substrate 1 (IRS-1) phosphorylation, but not Akt, mTOR, and p70S6K. Beef-protein-derived phenylalanine after resistance exercise appears more rapidly in circulation compared to milk ingestion [145], but both beef and milk ingestion increased the phosphorylation of mTOR complex 1 and p70S6K during post-exercise recovery.

In general, resistance exercise and BCAA exert both separate and combined effects on p70S6K phosphorylation. BCAA intake in combination with resistance training reduced the activation of signaling pathways resulting in protein breakdown. Whey protein leads to faster increases in blood BCAA concentrations and activates mTOR signaling in a dose-dependent manner.

One potential limitation regarding whey protein supplementation is related to the subsequent reduction in blood BCAA levels after acute ingestion, when compared with other protein sources. Research shows that blood BCAA levels return to baseline levels within 3 h of ingestion [100]. While the acute increase in muscle protein synthesis in response to whey protein ingestion is significant, as blood BCAA levels drop, additional amino acid oxidation can occur, which may limit protein synthesis and, rather, favor muscle protein breakdown [146]. Therefore, the concurrent ingestion of whey protein with another high-quality protein source that digests more slowly may offset this. A soy–dairy protein blend ingestion created a lower initial rise in blood BCAA compared to whey proteins but sustained elevated levels of blood amino acids later into recovery [146]. In a subsequent study, Reidy et al. [147] demonstrated that a soy–dairy protein blend and whey protein ingestion enhanced post-exercise amino acid transporter mRNA expression, transport into muscle, and myofibrillar protein synthesis. Furthermore, they showed that post-exercise ingestion of a protein blend resulted in a slightly prolonged net amino acid balance across the leg compared with whey protein.

Branched chain amino acid supplementation may also assist with delaying the onset of central fatigue. Central fatigue can be caused by a reduction in blood glucose due to a depletion of liver glycogen stores or an increase in 5-hydroxytryptamin (5-HT), also known as the neurotransmitter serotonin [148]. Newsholme et al. [149] were the first to identify the link between serotonin and the feeling of fatigue or sleepiness [150]. The synthesis of 5-HT occurs when the concentration of the free amino acid tryptophan increases, relative to other large neutral amino acids, like BCAAs [151]. During prolonged exercise, BCAAs may be oxidized for energy as glycogen stores deteriorate [152]. Additionally, plasma free fatty acids increase in the blood to meet the energy demands of the body. Since these free fatty acids competitively bind to the same sites on plasma albumin as tryptophan, the bound tryptophan is displaced and consequently increases the concentration of free tryptophan. Both the decrease in blood BCAA concentration and increase in free tryptophan causes an increase in tryptophan uptake across the blood–brain barrier and a subsequent increase in 5-HT synthesis. Therefore, the ability to control the ratio between blood BCAAs and free tryptophan may play an important role in delaying the onset of central fatigue. Hormoznejad and colleagues [153] conducted a systematic review of the literature and a meta-analysis to determine if BCAA supplementation was effective in delaying fatigue. After removing publications that did not meet the inclusion criteria, a total of 31 studies were included in the analysis. The analysis found that BCAA supplementation had a positive effect on various metabolites that are associated with fatigue, like lactate, ammonia, glucose, free fatty acids, and creatine kinase. Interestingly, however, no interaction effect was found between BCAA supplementation and the onset of central fatigue. The authors theorized this could have been due to the inconsistencies in central fatigue assessments used in the studies. The inconsistencies related to measuring central fatigue, as well as BCAA supplementation protocols, may provide an explanation of the mixed results. Indeed, another systematic review of BCAA supplementation identified that heterogeneity in the protocols was found to be a moderating variable and that caution should be exercised when interpreting the results [154]. Based on these analyses, there appears to be appropriate support for BCAA supplementation for the prevention of peripheral fatigue; however, more research may be needed to accurately quantify the effect BCAA supplementation has on delaying or reducing central fatigue.

## 5. Discussion

Over the past twenty years, supplementation strategies focused on nutrient timing have been extensively studied. However, establishing robust scientific recommendations remains challenging, due to inconsistent findings and the lack of studies dedicated to optimizing CHO, protein, and amino acid dosage and timing [155]. The current paper highlights the additive effects of resistance exercise and nutrient availability, and more practical approaches for nutritional supplementation strategies aimed at optimizing nutritional status during the pre-, intra-, and post-exercise period [156]. Given the major influences that nutrient quantity, quality, and timing have on muscle protein synthesis and degradation, as well as glycogen resynthesis, optimizing nutritional status throughout these periods may offer a sustained substrate response over the postprandial period. Since the optimization of blood EAA levels with minimal risk of gastric discomfort is desired, the ingestion of 3–18 g of EAA is recommended, depending on gastrointestinal tolerance [102]. During the exercise bout, ingestion of a mixed nutrient solution containing lower-concentration CHO (6–8%) and EAA (6–10 g) may attenuate the exercise-induced cortisol response, as well as increasing insulin and extracellular amino acid availability [25,31]. Immediately post-exercise, consumption of a whey/casein blend (20–30 g) may further enhance key signals promoting protein synthesis and muscle growth [55]. Finally, during the late post-exercise recovery period (2–4 h post-exercise), consumption of 1.2 g/kg CHO high glycemic index, combined with 0.4 g/kg PRO, may further alter the balance between hormone-mediated anabolic and catabolic activity, stimulating insulin and growth hormone elevations [37].

## 6. Conclusions

The aim of this review, in accordance with questions raised by the literature, was to establish whether there was any usefulness in nutritional supplementation during acute and chronic resistance exercise. Nutritional supplementation strategies modify the exercise-induced hormonal response. However, the mechanism(s) responsible for the acute response and chronic adaptations of these hormonal changes on skeletal muscle growth have remained relatively undefined. Collectively, the above studies support the notion that the magnitude and the duration of changes in nutrient status (i.e., CHO and amino acid availability) determine the anabolic effects on skeletal muscle, and that the inclusion of PRO with different digestive properties (whey and casein) may produce a sustained response over the postprandial period. Furthermore, the synergistic effect of mixed nutrient ingestion is a most effective nutritional strategy that maximizes the anabolic response of skeletal muscle. Therefore, it is recommended that strength and power athletes would benefit from a nutrient timing strategy that includes pre-, intra-, and/or post-exercise supplementation. The pre-exercise ingestion of whey PRO; CHO/EAA consumption during the resistance exercise bout; and/or the post-workout ingestion of a combined whey/casein blend may promote an environment aimed at optimizing exercise-induced skeletal muscle growth. While this review provides specific nutritional supplementation recommendations aimed at enhancing skeletal muscle anabolism, further research is warranted to elucidate the time course and magnitude of changes in muscle protein metabolism following nutritive intervention in strength and power athletes.

## Figures and Tables

**Table 1:** Literature examining nutritional supplementation and acute resistance exercise.

Study	Subjects	Protocol	Intervention	Time Course	Measures	Outcomes
Biolo et al. [48]	6 untrained M	Lower-body 4 exercises; 4–5 sets × 8–10 reps; 75% 1-RM	Infusion of balanced AA mix 0.15 g·kg^−1^·h^−1^	3 h at rest 3 h post-exercise	Muscle biopsies A/V blood samples	MPS greater after RE (>200%) than at rest (~150%). MPB not significantly different after either condition. AA transport increased 30–100% post-ex compared to resting condition. Suggested ↑ AA availability post-ex mediates anabolic response.
Bird et al. [31,42]	32 untrained M (18–29 yrs)	Complete-body 8 exercises; 3 sets × 10 reps; 75% 1-RM	Four groups: EAA (6 g) CHO (6%) CHO+EAA PLA	~625 mL beverage Ingested during ex bout. Fluid volume 8.5 mL/kg	Blood sampling: 15 min intervals: 0, 15, 30, 45, 60, 75, and 90 min Biochemical: Cortisol; Insulin; Testosterone; Glucose Urine: 3-MH	Ingestion of liquid CHO, EAA, CHO+EAA solution during exercise bout blunted exercise-induced cortisol response, CHO+EAA consumption resulting in significantly ↓ 3-MH excretion.
Borsheim et al. [18]	6 active ind. 3 M/3 F	Lower-body 2 exercises; 18 sets × 8–10 reps; 80% 1-RM	6 g EAA × 2	425 mL bolus EAA ingestion, 1 h and 2 h post-ex	Muscle biopsies 0.5, 1.5, 2.5, and 4 h post-ex Femoral A/V blood 15 over 7 h	Net muscle protein balance ↑ following ingestion at both 1 and 2 h post-ex. The response of net balance was about twice the response to 6 g of mixed AA (Miller et al. [36]). NEAA are not required to stimulate MPS. Suggested dose-dependent effect of EAA ingestion on MPS following RE.
Borsheim, Aarsland and Wolfe [49]	8 healthy ind. 5 M/3 F	Knee extensor exercise 10 sets × 8 reps; 80% 1-RM	Two conditions: CHO (100 g) PAAC (17.5 g Whey + 4.9 g AA + 77.4 g CHO)	Fluid volume of 590 mL ingested 1 h post-ex	Muscle biopsies 0.5, 1, 2, 4 h post-ex Femoral A/V blood 17 over 6 h	PAAC ↑ MPS to a greater extent than CHO alone. Response appeared to consist of two phases, one rapid acute phase followed by smaller delayed phase ~90 min after ingestion. Suggested that response due to insulin effect and sustained elevation in AA. Suggested that addition of whey to CHO+AA extends anabolic effect, lasting beyond first hour after intake.
Borsheim et al. [14]	16 healthy ind. 10 M/6 F	Knee extensor exercise 10 sets × 8 reps; 80% 1-RM	Two groups: Placebo (PLA) CHO (100 g)	Beverage ingested 1 h post-ex	Muscle biopsies: 0.5, 1, 2, 4 h post-ex Femoral A/V blood 16 over 6 h	CHO ingestion resulted in significant ↑ in glucose and insulin concentration, no change in PLA. Corresponded with no change in net muscle protein balance in PLA, whereas net balance was improved following CHO ingestion. This response was attributed to a progressive decrease in MPB. Overall improvement small compared to AA ingestion. Suggested AA necessary for maximal anabolic response.
Hulmi et al. [50]	10 trained M	Lower-body 3 exercises; 3–5 sets × 1–10 reps; RM load	Two conditions: Placebo (PLA) PRO (17.5 g whey + 7.5 g casein) PRO+CHO (25 g)	500 mL beverage PLA or PRO ingested 30 min pre-ex PLUS PRO+CHO ingested 5 min post-ex	Blood sampling: 0, 0.5, 1, and 2 h post-ex Hormones: Cortisol; Testosterone; Insulin; Growth Hormone	PRO intake 30 pre-ex ↑ insulin by 51.6% 5 min post-ex. However, testosterone and growth hormone were significantly ↓ 5 min post-ex compared to PLA. Concluded that PRO consumption 30 min before RE will provide a more anabolic hormonal environment by ↑ insulin and possibly testosterone uptake.
Koopman et al. [51]	8 untrained M	Lower-body 2 exercises; 16 sets × 8 reps; 80% 1-RM	Three conditions: CHO: 50 g CHO+PRO: 50 g CHO+Whey: 33.3 g CHO+PRO+Leu: 50 g CHO + 33.3 g whey + 16.6 g Leu	Post-ex ingestion Dosage in g/L Fluid volume 3 mL·kg^−1^·30 min^−1^	Muscle biopsies: 0 and 6 h post-ex A/V blood samples 16 over 6 h	Over the 6 h post-ex period, whole-body protein breakdown ↓, and whole-body protein breakdown ↑ in CHO+PRO and CHO+PRO+Leu conditions compared with CHO. This corresponded with ↑ insulin concentrations in CHO+PRO and CHO+PRO+Leu conditions than CHO. FSR ↑ in CHO+PRO+Leu compared with CHO. Concluded that co-ingestion of PRO and Leu stimulates MPS and optimizes protein balance compared with CHO only.
Miller et al. [36]	10 healthy ind. 6 M/4 F	Lower-body 2 exercises; 18 sets × 8–10 reps; 75% 1-RM	Three conditions: CHO: ~35 g 6 g AA mix: 2.8 g EAA/3.2 g NEEA MIX: ~35 g CHO + 6 g AA	Beverage ingested 1 h and 2 h post-ex Composition adjusted according to body mass	Muscle biopsies: 0.5, 1.5, 2.5, and 3.5 h post-ex Femoral A/V blood: 8 over 4 h	Ingestion of CHO+AA (MIX) and AA significantly ↑ MPS compared to CHO; however, no differences reported between MIX and AA. Ingestion of the second drink 1 h after the first drink stimulated a similar response as to the first drink. Concluded that combined effects of CHO and AA ingestion following RE reflects sum of their individual effects. Ingestion of only EAA is required to stimulate MPS.
Rasmussen et al. [22]	6 untrained 3 M/3 F	Lower-body 2 exercises; 18 sets × 8 reps; 80% 1-RM	Two conditions: Placebo (PLA) EAC: 6 g EAA + 35 g CHO	PLA 1 h post-ex + EAC 3 h post-ex EAC 1 h post-ex + PLA 3 h post-ex	Muscle biopsies: 1, 2, and 4 h post-ex A/V blood samples: 11 over 7 h period	EAC ↑ glucose and insulin concentration, MPS and muscle protein net balance at both 1 h and 3 h. No change was reported for PLA. MPB was unaffected regardless of when EAC or PLA was ingested. Timing of EAC ingestion did not affect the response MPS or muscle protein net balance, no significant difference reported between 1 h and 3 h.
Roy et al. [52]	8 young M (20–25 yr)	Single-leg knee extensor exercise; 8 sets × 10 reps; 85% 1-RM. One leg performed exercise, while other leg served as control	Two conditions: Placebo (PLA) CHO (1 g/kg)	Immediately and 60 min post-ex	Muscle biopsies: 0 and 10 h post-ex A/V blood samples: 14 over 10 h period 24 h urine collection	CHO ingestion resulted in significant ↑ in glucose and insulin concentrations, which corresponded with reduction in 3-MHIS and urea excretion. This was interpreted as a reduction in MPB. Suggested that net effect was anabolic and would result in more positive net muscle protein balance.
Roy et al. [44]	10 young M (18–21 yr)	Whole-body 9 exercises; 3 sets × 10 reps; 80% 1-RM	Three conditions: Placebo (PLA) CHO (1 g/kg) CHO/PRO/FAT (1 g/kg)	Immediately and 60 min post-ex	A/V blood samples: 13 over ~6 h period	NOLD was ~41% and ~33% greater for CHO/PRO/FAT and CHO compared with PLA at 4 h post-ex. CHO/PRO/FAT ingestion led to similar ↑ in glucose and insulin to CHO (Roy et al. 1997). No significant differences for 3-MHIS between conditions. However, the authors note that there was a directional change that was lower for the supplement conditions compared to PLA.
Tarpenning et al. [53]	5 older M (55–64 yr)	Complete-body 9 exercises; 3 sets × 10 reps; 75% 1-RM	Two conditions: Placebo (PLA) CHO (6%)	Beverages ingested during exercise bout Fluid volume 8.5 mL/kg	Biochemical: Cortisol Free testosterone Glucose	PLA displayed significant ↑ of 67% in plasma cortisol levels. Time of peak cortisol concentration corresponded with non-significant change in glucose levels of 8%. CHO trial resulted in a blunted cortisol response (non-significant change), corresponded with significant ↑ of 37% in glucose levels. Suggested that CHO-induced modification of cortisol response may modulate ↓ in neuroendocrine function in older individuals.
Titpon et al. [17]	6 untrained 3 M/3 F	Lower-body 4 exercises; 4–5 sets × 8–10 reps; 75% 1-RM	Three conditions: Placebo (PLA) 40 g mixed AA 40 g EAA	60 min post-ex 100 mL every 18–20 min until 4 h post-ex. Fluid volume 1 liter	Muscle biopsy: 4.5 h post-ex A/V blood samples: 5 over 7.5 h period	Both MAA and EAA produced hyperaminoacidemia, thereby ↑ muscle protein net balance similar to levels attained by infusion. Concluded that it is not necessary to include NEAA in formulation to elicit anabolic response, as muscle protein net balance was similar for MAA and EAA.
Tipton et al. [54]	6 active ind. 3 M/3 F	Lower-body 2 exercises; 18 sets × 8 reps; 80% 1-RM	Two conditions: Placebo (PLA) EAC: 6 g EAA + 35 g CHO	500 mL bolus EAC ingestion: PRE-ex condition POST-ex condition	Muscle biopsies: −60, 0, 60, and 120 min A/V blood samples: 16 over 4 h period	AA delivery and stimulation of MPS was significantly greater in PRE-ex than POST-ex. Concluded that effectiveness of EAC ingestion consumed PRE is superior to when consumed after RE. The combination of ↑ blood flow and AA availability maximizes the anabolic response to RE.
Tipton et al. [19]	7 healthy ind. 4 M/3 F	Knee extensor exercise 8 sets × 8 reps; 80% 1-RM	Two conditions: Resting (REST) ES: 15 g EAA × 2 +RE	350 mL bolus EAC ingestion Pre-ex and 1 h post-ex	Muscle biopsies: 5 over 24 h period A/V blood samples: 37 over 24 h period	FSR over full 24 h period was ~40% greater in ES. Suggested that changes in net muscle protein balance over short period (3 h) are representative of changes in net balance over 24 h period. MPS stimulated by RE and EAA ingestion is additive to balance that occurs in REST condition.
Tipton et al. [55]	23 healthy M/F	Knee extensor exercise 10 sets × 8 reps; 80% 1-RM	Three groups: Placebo (PLA) Casein (CS): 20 g Whey (WH): 20 g	Fluid volume of 300 mL ingested 1 h post-ex	Muscle biopsies: −0.5, 1, 2, 5 h post-ex A/V blood samples: 17 over 6 h	Both CS and WH resulted in positive AA balance, initiative of net MPS. Pattern of AA appearance peaked earlier and at greater magnitude for WH than CS. Insulin concentrations mirrored this response, ↑ more rapidly WH than CS. Suggested that differences in digestive properties contributed to pattern of response. Concluded that post-ex ingestion of whole proteins may be effective in ↑ muscle size following resistance training.

Abbreviations: M = Male; F = Female; RE = Resistance exercise; RM = Repetition maximum; reps = Repetitions; ↑ = Increase; ↓ = Decrease; MPS = Muscle protein synthesis; MPB = Muscle protein breakdown; AA = Amino acids; EAA = Essential amino acids; NEAA = Non-essential amino acids CHO = Carbohydrate; PRO = Protein; A/V = Arteriovenous; Pre-ex = Pre-exercise; Post-ex = Post-exercise; NOLD = Non-oxidative leucine disposal; FSR = Fractional muscle protein synthetic rate; Cr = Creatine; FFM = Fat free mass; Leu = Leucine.

**Table 2:** Literature examining nutritional supplementation and resistance training.

Study	Subjects	Protocol	Intervention	Time Course	Measures	Outcomes
Andersen et al. [24]	22 untrained M	Resistance Training: 14 weeks 3 d/wk; Lower-body; 3 exercises 3–4 sets × 4–15 reps; RM loads	Two groups: PRO (16.6 g whey + 2.8 g casein + 2.8 g egg white +2.8 g L-glutamine) CHO (25 g)	Fluid volume of 500 mL ingested immediately pre- and post-ex Morning ingestion on non-training days	Pre- and post- training: Muscle fiber CSA Muscular strength	PRO ingestion ↑ type I (18%) and type II (26%) CSA, which corresponded with 9% ↑ in vertical jump performance. No significant change reported for CHO. Interestingly, similar ↑ in peak torque were reported for both groups. Concluded that PRO ingestion has minor advantage over CHO in muscular properties (CSA and mechanical function).
Burke et al. [56]	36 trained M (18–31 yr)	Resistance Training: 12 weeks 4 d/wk; 2 d split (Upper/Lower-body) 4–5 sets × 6–12 reps; RM loads	Three groups: Whey: 1.2 g/kg Whey+Cr: 1.2 g/kg + 0.1 g/kg Cr PLA	Four equal servings across the day	Pre- and post- training: Body composition Muscular strength	Supplementation with combination of Whey+Cr resulted in greater increases in lean tissue mass as determined by DEXA and bench press 1-RM strength than for those who supplemented with only whey or PLA.
Chromiak et al. [57]	41 healthy M (18–35 yr)	Resistance Training: 10 weeks 4 d/wk; 2 d split (Upper/Lower-body) 3–4 sets × 3–10 reps; RM loads	Two groups: SUPP: 13 g whey + 4.9 g AA + 3 g Cr + 76 g CHO CHO: (92 g)	Beverage ingested post-ex	Pre- and post- training: Body composition Muscular strength Muscular power Anaerobic capacity	Post-ex SUPP did not result in greater improvements in performance variables compared with CHO. However, a trend (*p* = 0.07) was reported towards a greater ↑ in FFM in the SUPP group.
Cribb et al. [58]	13 M recreational bodybuilders	Resistance Training: 10 weeks 3 d/wk; Upper/Lower Split Wks 1–2: Preparatory phase, 10–8 RM Wks 3–6: Overload phase 1, 6 RM Wks 7–10: Overload phase 2, 4 RM	Two groups: WI: Whey isolate C: Casein	Ingested 1.5 g/kg/d dose divided into 4 smaller equal serves: Serve 1: Breakfast Serve 2: Lunch Serve 3: Post-workout Serve 4: Evening	Pre- and post- training: Body composition Muscular strength Plasma glutamine	WI group demonstrated a significantly ↑ strength, LBM, and significant ↓ in fat mass compared to the C group during an intense 10 wk resistance training program. Neither supplement influenced plasma glutamine.
Cribb et al. [59]	33 M recreational bodybuilders	Resistance Training: 10 weeks Whole-body; 3 exercises Wks 1–2: Preparatory phase, 10 RM Wks 3–6: Overload phase 1, 8–6 RM Wks 7–10: Overload phase 2, 6–4 RM	Three groups: PRO: Whey PRO-CHO: 50% whey; 50% glucose Cr-PRO-CHO: As above, plus creatine monohydrate	Ingested 1.5 g/kg/d dose divided into 3 smaller equal serves: Serve 1: Morning Serve 2: Post-workout (or afternoon on non-training days) Serve 3: Before sleep Cr supplements: 1-wk loading 0.3 g/kg/d, followed by 0.1 g/kg/d	Pre- and post- training: Body composition Muscular strength Muscle fiber CSA	Cr-containing supplement (Cr-PRO-CHO) demonstrated greater gains in 1 RM strength in three exercises, and these improvements were supported by greater hypertrophy response that was apparent at three different levels: LBM, muscle fiber CSA, and contractile protein content.
Ratamess et al. [60]	17 trained M	Resistance Training: 4-week over-reaching program; 4 d/wk Total-body 2 weeks base: 3 sets × 8–10 reps; 80% 1-RM; 2 weeks high-intensity: 3 sets × 8–10 reps; RM load	Two groups: Placebo (PLA) AA mixture (0.4 g/kg)	PLA or AA capsules divided into three daily doses	Pre- and post- training: Body composition Muscular strength Muscular power Muscular endurance	AA supplementation was effective in attenuating reductions in strength and power output during 4 weeks of resistance training over-reaching. Furthermore, it appears that following an initial phase of high-volume, moderate-intensity RE with a phase of higher-intensity, moderate-intensity RE is effective in ↑ muscular strength in resistance-trained males.
Rankin et al. [61]	19 untrained M (18–25 yr)	Resistance Training: 10 weeks 3 d/wk; Whole-body; 7 exercises 3–5 sets × 3–12 reps; 55–97% 1-RM	Two groups: MILK (0.92 g/kg CHO + 0.21 g/kg PRO + 0.06 g/kg FAT) CHO (125 g/kg)	Beverage ingested immediately post-ex	Pre- and post- training: Body composition Muscular strength Hormone levels Energy expenditure	The type of beverage consumed, MILK or CHO, did not have a significant effect on any performance measure. A trend was reported in the MILK group for body mass (*p* = 0.10) and fat free soft tissue (*p* = 0.13). Concluded that adaptations to resistance training were similar whether nutritional supplement of MILK or CHO was ingested immediately post-ex.
Schoenfeld et al. [62]	21 M strength trained	Resistance Training: 10 weeks 3 d/wk; Whole-body; 3 sets × 10 reps; 75% 1-RM	Two groups: PRE-SUPP POST-SUPP Supp: 24 g PRO + 1 g CHO	Ingested immediately pre- or post-exercise	Pre-, mid-, and post-training: Body composition Muscle thickness Muscular strength	No differences between groups across all measures. Concluded that the proposed post-exercise ‘anabolic window’ may not be as important as ensuring adequate protein intake. The interval for protein intake to stimulate MPS may be as wide as several hours post-training depending on intake timing.
Tarpenning et al. [15]	8 young M (18–25 yr)	Resistance Training: 12 weeks 3 d/wk; Whole-body; 3sets × 10 reps; 75% 1-RM	Two groups: Placebo (PLA) CHO (6%)	Beverages ingested during exercise bout Fluid volume 8.5 mL/kg	Pre- and post- training: Muscle fiber CSA Body composition Muscular strength Hormone levels	CHO ingestion blunted exercise-induced cortisol response (av. ↓ of 4.1%) compared to PLA (av. ↑ of 81.9%). CHO group displayed greater gains in type I and type II CSA. Reduction in cortisol response related to ↑ in muscle hypertrophy. Suggested that CHO-induced modification of cortisol response positively impacts skeletal muscle hypertrophic adaptation.
Taylor et al. [63]	16 F NCAA DivIII basketball players	Resistance Training: 8 weeks 3 d/wk; Whole-body; 3 sets × 12 RM	Two groups: WP: Whey (24 g) MD: CHO (24 g)	Pre- and post-resistance training	Pre- and post- training: Body composition Muscular strength Performance testing	WP group, significant change in body composition (lean mass ↑ 1.4 kg; fat mass ↓ 1.0 kg); greater gains in 1 RM bench and improved pro agility time compared to MD. Concluded that 8 weeks of whey protein supplementation improved body composition and increased performance variables in previously trained female athletes.
Kerksick et al. [64]	36 resistance-trained M (18–50 yr)	Resistance Training: 10 weeks 4 d/wk; Split body part 2 x upper-body; 2 x lower-body; 3 sets × 6–10 RM	Three groups: WBG: Whey + BCAAs + L-glutamine WC: Whey + casein PLA: CHO	Ingested supplement within 2 hrs post-workout on training days	Pre-, mid-, and post-training: Body composition Muscular strength Muscular endurance Anaerobic capacity	Whey + casein group showed greatest ↑ in lean mass. 1 RM for bench press and leg press significantly ↑ in all groups. Concluded that whey and casein protein supplementation significantly improve body composition via increases in lean mass.

Abbreviations: M = Male; F = Female; RE = Resistance exercise; RM = Repetition maximum; reps = Repetitions; DEXA = Duel energy x-ray absorptiometry; ↑ = Increase; ↓ = Decrease; MPS = Muscle protein synthesis; AA = Amino acids; EAA = Essential amino acids; Leu = Leucine; CHO = Carbohydrate; PRO = Protein; Pre-ex = Pre-exercise; Post-ex = Post-exercise; CSA = Cross-sectional area; Cr = Creatine; FFM = Fat free mass.

## References

[1] Bird S.P., Tarpenning K.M. (2004). Influence of circadian time structure on acute hormonal responses to a single bout of heavy-resistance exercise in weight-trained men. Chronobiol. Int..

[2] Gotshalk L.A., Loebel C.C., Nindl B.C., Putukian M., Sebastianelli W.J., Newton R.U., Hakkinen K., Kraemer W.J. (1997). Hormonal responses of multiset versus single-set heavy-resistance exercise protocols. Can. J. Appl. Physiol..

[3] Hakkinen K., Pakarinen A. (1995). Acute hormonal responses to heavy resistance exercise in men and women at different ages. Int. J. Sports Med..

[4] Izquierdo M., Ibanez J., Calbet J.A., Navarro-Amezqueta I., Gonzalez-Izal M., Idoate F., Hakkinen K., Kraemer W.J., Palacios-Sarrasqueta M., Almar M. (2009). Cytokine and hormone responses to resistance training. Eur. J. Appl. Physiol..

[5] Kraemer W.J., Marchitelli L., Gordon S.E., Harman E., Dziados J.E., Mello R., Frykman P., McCurry D., Fleck S.J. (1990). Hormonal and growth factor responses to heavy resistance exercise protocols. J. Appl. Physiol..

[6] Raastad T., Bjoro T., Hallen J. (2000). Hormonal responses to high- and moderate-intensity strength exercise. Eur. J. Appl. Physiol..

[7] Ahtiainen J.P., Pakarinen A., Alen M., Kraemer W.J., Hakkinen K. (2003). Muscle hypertrophy, hormonal adaptations and strength development during strength training in strength-trained and untrained men. Eur. J. Appl. Physiol..

[8] Kraemer W.J., Häkkinen K., Newton R.U., Nindl B.C., Volek J.S., McCormick M., Gotshalk L.A., Gordon S.E., Fleck S.J., Campbell W.W. (1999). Effects of heavy-resistance training on hormonal response patterns in younger vs. older men. J. Appl. Physiol..

[9] Kraemer W.J., Ratamess N.A. (2005). Hormonal responses and adaptations to resistance exercise and training. Sports Med..

[10] McCall G.E., Byrnes W.C., Fleck S.J., Dickinson A., Kraemer W.J. (1999). Acute and chronic hormonal responses to resistance training designed to promote muscle hypertrophy. Can. J. Appl. Physiol..

[11] Staron R.S., Karapondo D.L., Kraemer W.J., Fry A.C., Gordon S.E., Falkel J.E., Hagerman F.C., Hikida R.S. (1994). Skeletal muscle adaptations during early phase of heavy-resistance training in men and women. J. Appl. Physiol..

[12] Florini J.R. (1987). Hormonal control of muscle growth. Muscle Nerve.

[13] Kraemer W.J. (1988). Endocrine responses to resistance exercise. Med. Sci. Sports Exerc..

[14] Borsheim E., Cree M.G., Tipton K.D., Elliott T.A., Aarsland A., Wolfe R.R. (2004). Effect of carbohydrate intake on net muscle protein synthesis during recovery from resistance exercise. J. Appl. Physiol..

[15] Tarpenning K.M., Wiswell R.A., Hawkins S.A., Marcell T.J. (2001). Influence of weight training exercise and modification of hormonal response on skeletal muscle growth. J. Sci. Med. Sport.

[16] Thyfault J.P., Carper M.J., Richmond S.R., Hulver M.W., Potteiger J.A. (2004). Effects of liquid carbohydrate ingestion on markers of anabolism following high-intensity resistance exercise. J. Strength Cond. Res..

[17] Tipton K.D., Ferrando A.A., Phillips S.M., Doyle D., Wolfe R.R. (1999). Postexercise net protein synthesis in human muscle from orally administered amino acids. Am. J. Physiol. Endocrinol. Metabol..

[18] Borsheim E., Tipton K.D., Wolf S.E., Wolfe R.R. (2002). Essential amino acids and muscle protein recovery from resistance exercise. Am. J. Physiol. Endocrinol. Metabol..

[19] Tipton K.D., Borsheim E., Wolf S.E., Sanford A.P., Wolfe R.R. (2003). Acute response of net muscle protein balance reflects 24-h balance after exercise and amino acid ingestion. Am. J. Physiol. Endocrinol. Metabol..

[20] Bird S.P., Mabon T., Pryde M., Feebrey S., Cannon J. (2013). Triphasic multinutrient supplementation during acute resistance exercise improves session volume load and reduces muscle damage in strength-trained athletes. Nutr. Res..

[21] Kraemer W.J., Hatfield D.L., Spiering B.A., Vingren J.L., Fragala M.S., Ho J.-Y., Volek J.S., Anderson J.M., Maresh C.M. (2007). Effects of a multi-nutrient supplement on exercise performance and hormonal responses to resistance exercise. Eur. J. Appl. Physiol..

[22] Rasmussen B.B., Tipton K.D., Miller S.L., Wolf S.E., Wolfe R.R. (2000). An oral essential amino acid-carbohydrate supplement enhances muscle protein anabolism after resistance exercise. J. Appl. Physiol..

[23] Volek J.S. (2004). Influence of nutrition on responses to resistance training. Med. Sci. Sports Exerc..

[24] Andersen L.L., Tufekovic G., Zebis M.K., Crameri R.M., Verlaan G., Kjaer M., Suetta C., Magnusson P., Aagaard P. (2005). The effect of resistance training combined with timed ingestion of protein on muscle fiber size and muscle strength. Metab.Clin. Exp..

[25] Bird S.P., Tarpenning K.M., Marino F.E. (2006). Independent and combined effects of liquid carbohydrate/essential amino acid ingestion on hormonal and muscular adaptations following resistance training in untrained men. Eur. J. Appl. Physiol..

[26] King A., Kwan K., Jukic I., Zinn C., Helms E. (2024). Fueling for and recovering from resistance training: The periworkout nutrition practices of competitive powerlifters. Nutrition.

[27] Rasmussen B.B., Phillips S.M. (2003). Contractile and nutritional regulation of human muscle growth. Exerc. Sport Sci. Rev..

[28] Rennie M.J., Tipton K.D. (2000). Protein and amino acid metabolism during and after exercise and the effects of nutrition. Annu. Rev. Nutr..

[29] Rennie M.J., Wackerhage H., Spangenburg E.E., Booth F.W. (2004). Control of the size of the human muscle mass. Annu. Rev. Physiol..

[30] Bird S.P., Tarpenning K.M., Marino F.E. (2005). Designing resistance training programmes to enhance muscular fitness: A review of the acute programme variables. Sports Med..

[31] Bird S.P., Tarpenning K.M., Marino F.E. (2006). Effects of liquid carbohydrate/essential amino acid ingestion on acute hormonal response during a single bout of resistance exercise in untrained men. Nutrition.

[32] Kraemer W.J., Volek J.S., Bush J.A., Putukian M., Sebastianelli W.J. (1998). Hormonal responses to consecutive days of heavy-resistance exercise with or without nutritional supplementation. J. Appl. Physiol..

[33] Smilios I., Pilianidis T., Karamouzis M., Tokmakidis S.P. (2003). Hormonal responses after various resistance exercise protocols. Med. Sci. Sports Exerc..

[34] Williams A.G., Ismail A.N., Sharma A., Jones D.A. (2002). Effects of resistance exercise volume and nutritional supplementation on anabolic and catabolic hormones. Eur. J. Appl. Physiol..

[35] Phillips S.M. (2009). Physiologic and molecular bases of muscle hypertrophy and atrophy: Impact of resistance exercise on human skeletal muscle (protein and exercise dose effects). Appl. Physiol. Nutr. Metab..

[36] Miller S.L., Tipton K.D., Chinkes D.L., Wolf S.E., Wolfe R.R. (2003). Independent and combined effects of amino acids and glucose after resistance exercise. Med. Sci. Sports Exerc..

[37] Chandler R.M., Byrne H.K., Patterson J.G., Ivy J.L. (1994). Dietary supplements affect the anabolic hormones after weight-training exercise. J. Appl. Physiol..

[38] Fafournoux P., Bruhat A., Jousse C. (2000). Amino acid regulation of gene expression. Biochem. J.

[39] Hamel F.G., Upward J.L., Siford G.L., Duckworth W.C. (2003). Inhibition of proteasome activity by selected amino acids. Metab.Clin. Exp..

[40] Varillas-Delgado D., Del Coso J., Gutiérrez-Hellín J., Aguilar-Navarro M., Muñoz A., Maestro A., Morencos E. (2022). Genetics and sports performance: The present and future in the identification of talent for sports based on DNA testing. Eur. J. Appl. Physiol..

[41] Elia M., Carter A., Bacon S., Winearls C.G., Smith R. (1981). Clinical usefulness of urinary 3-methylhistidine excretion in indicating muscle protein breakdown. Br. Med. J..

[42] Bird S.P., Tarpenning K.M., Marino F.E. (2006). Liquid carbohydrate/essential amino acid ingestion during a short-term bout of resistance exercise suppresses myofibrillar protein degradation. Metab.Clin. Exp..

[43] Candow D.G., Burke N.C., Smith-Palmer T., Burke D.G. (2006). Effect of whey and soy protein supplementation combined with resistance training in young adults. Int. J. Sport Nutr. Exerc. Metabol..

[44] Roy B.D., Fowles J.R., Hill R., Tarnopolsky M.A. (2000). Macronutrient intake and whole body protein metabolism following resistance exercise. Med. Sci. Sports Exerc..

[45] Garlick P.J., McNurlan M.A., Ballmer P.E. (1991). Influence of dietary protein intake on whole-body protein turnover in humans. Diabetes Care.

[46] Burke L.M., Hawley J.A. (2006). Fat and carbohydrate for exercise. Curr. Opin. Clin. Nutr. Metab. Care..

[47] Lambert C.P., Frank L.L., Evans W.J. (2004). Macronutrient considerations for the sport of bodybuilding. Sports Med..

[48] Biolo G., Tipton K.D., Klein S., Wolfe R.R. (1997). An abundant supply of amino acids enhances the metabolic effect of exercise on muscle protein. Am. J. Physiol. Endocrinol. Metabol..

[49] Borsheim E., Aarsland A., Wolfe R.R. (2004). Effect of an amino acid, protein, and carbohydrate mixture on net muscle protein balance after resistance exercise. Int. J. Sport Nutr. Exerc. Metabol..

[50] Hulmi J.J., Volek J.S., Selanne H., Mero A.A. (2005). Protein ingestion prior to strength exercise affects blood hormones and metabolism. Med. Sci. Sports Exerc..

[51] Koopman R., Wagenmakers A.J., Manders R.J., Zorenc A.H., Senden J.M., Gorselink M., Keizer H.A., van Loon L.J. (2005). Combined ingestion of protein and free leucine with carbohydrate increases postexercise muscle protein synthesis in vivo in male subjects. Am. J. Physiol. Endocrinol. Metabol..

[52] Roy B.D., Tarnopolsky M.A., MacDougall J.D., Fowles J., Yarasheski K.E. (1997). Effect of glucose supplement timing on protein metabolism after resistance training. J. Appl. Physiol..

[53] Tarpenning K.M., Hawkins S.A., Wiswell R.A. (2003). CHO-induced blunting of cortisol response to weightlifting exercise in resistance-trained older men. Eur. J. Sport Sci..

[54] Tipton K.D., Rasmussen B.B., Miller S.L., Wolf S.E., Owens-Stovall S.K., Petrini B.E., Wolfe R.R. (2001). Timing of amino acid-carbohydrate ingestion alters anabolic response of muscle to resistance exercise. Am. J. Physiol. Endocrinol. Metabol..

[55] Tipton K.D., Elliott T.A., Cree M.G., Wolf S.E., Sanford A.P., Wolfe R.R. (2004). Ingestion of casein and whey proteins result in muscle anabolism after resistance exercise. Med. Sci. Sports Exerc..

[56] Burke D.G., Chilibeck P.D., Davidson K.S., Candow D.G., Farthing J., Smith-Palmer T. (2001). The effect of whey protein supplementation with and without creatine monohydrate combined with resistance training on lean tissue mass and muscle strength. Int. J. Sport Nutr. Exerc. Metabol..

[57] Chromiak J.A., Smedley B., Carpenter W., Brown R., Koh Y.S., Lamberth J.G., Joe L.A., Abadie B.R., Altorfer G. (2004). Effect of a 10-week strength training program and recovery drink on body composition, muscular strength and endurance, and anaerobic power and capacity. Nutrition.

[58] Cribb P.J., Williams A.D., Carey M.F., Hayes A. (2006). The effect of whey isolate and resistance training on strength, body composition, and plasma glutamine. Int. J. Sport Nutr. Exerc. Metabol..

[59] Cribb P.J., Williams A.D., Hayes A. (2007). A creatine-protein-carbohydrate supplement enhances responses to resistance training. Med. Sci. Sports Exerc..

[60] Ratamess N.A., Kraemer W.J., Volek J.S., Rubin M.R., Gomez A.L., French D.N., Sharman M.J., McGuigan M.M., Scheett T., Hakkinen K. (2003). The effects of amino acid supplementation on muscular performance during resistance training overreaching. J. Strength Cond. Res..

[61] Rankin J.W., Goldman L.P., Puglisi M.J., Nickols-Richardson S.M., Earthman C.P., Gwazdauskas F.C. (2004). Effect of post-exercise supplement consumption on adaptations to resistance training. J. Am. Coll. Nutr..

[62] Schoenfeld B.J., Aragon A.A., Wilborn C., Urbina S.L., Hayward S.E., Krieger J. (2017). Pre- versus post-exercise protein intake has similar effects on muscular adaptations. PeerJ.

[63] Taylor L.W., Wilborn C., Roberts M.D., White A., Dugan K. (2016). Eight weeks of pre- and postexercise whey protein supplementation increases lean body mass and improves performance in Division III collegiate female basketball players. Appl. Physiol. Nutr. Metab..

[64] Kerksick C.M., Rasmussen C.J., Lancaster S.L., Magu B., Smith P., Melton C., Greenwood M., Almada A.L., Earnest C.P., Kreider R.B. (2006). The effects of protein and amino acid supplementation on performance and training adaptations during ten weeks of resistance training. J. Strength Cond. Res..

[65] Conley M.S., Stone M.H. (1996). Carbohydrate ingestion/supplementation for resistance exercise and training. Sports Med..

[66] Haff G.G., Lehmkuhl M.J., McCoy L.B., Stone M.H. (2003). Carbohydrate supplementation and resistance training. J. Strength Cond. Res..

[67] Robergs R.A., Pearson D.R., Costill D.L., Fink W.J., Pascoe D.D., Benedict M.A., Lambert C.P., Zachweija J.J. (1991). Muscle glycogenolysis during differing intensities of weight-resistance exercise. J. Appl. Physiol..

[68] Tesch P.A., Ploutz-Snyder L.L., Ystrom L., Castro M.J., Dudley G.A. (1998). Skeletal muscle glycogen loss evoked by resistance exercise. J. Strength Cond. Res..

[69] Pascoe D.D., Costill D.L., Fink W.J., Robergs R.A., Zachwieja J.J. (1993). Glycogen resynthesis in skeletal muscle following resistive exercise. Med. Sci. Sports Exerc..

[70] Roy B.D., Tarnopolsky M.A. (1998). Influence of differing macronutrient intakes on muscle glycogen resynthesis after resistance exercise. J. Appl. Physiol..

[71] Haff G.G., Koch A.J., Potteiger J.A., Kuphal K.E., Magee L.M., Green S.B., Jakicic J.J. (2000). Carbohydrate supplementation attenuates muscle glycogen loss during acute bouts of resistance exercise. Int. J. Sport Nutr. Exerc. Metabol..

[72] Lambert C.P., Flynn M.G., Boone J.B., Michaud T.J., Rodriguez-Zayas J. (1991). Effects of carbohydrate feeding on multiple-bout resistance exercise. J. Appl. Sport Sci. Res..

[73] Haff G.G., Stone M.H., Warren B.J., Keith R., Johnson R.L., Nieman D.C., Williams F., Kirksey K.B. (1999). The effect of carbohydrate supplementation on multiple sessions and bouts of resistance exercise. J. Strength Cond. Res..

[74] Williams A.G., van den Oord M., Sharma A., Jones D.A. (2001). Is glucose/amino acid supplementation after exercise an aid to strength training?. Br. J. Sports Med..

[75] Kraemer W.J., Spiering B.A., Volek J.S., Ratamess N.A., Sharman M.J., Rubin M.R., French D.N., Silvestre R., Hatfield D.L., Van Heest J.L. (2006). Androgenic responses to resistance exercise: Effects of feeding and L-carnitine. Med. Sci. Sports Exerc..

[76] Arfvidsson B., Zachrisson H., Moller-Loswick A.C., Hyltander A., Sandstrom R., Lundholm K. (1991). Effect of systemic hyperinsulinemia on amino acid flux across human legs in postabsorptive state. Am. J. Physiol. Endocrinol. Metabol..

[77] Moller-Loswick A.C., Zachrisson H., Hyltander A., Korner U., Matthews D.E., Lundholm K. (1994). Insulin selectively attenuates breakdown of nonmyofibrillar proteins in peripheral tissues of normal men. Am. J. Physiol. Endocrinol. Metabol..

[78] Koch A.J., Potteiger J.A., Chan M.A., Benedict S.H., Frey B.B. (2001). Minimal influence of carbohydrate ingestion on the immune response following acute resistance exercise. Int. J. Sport Nutr. Exerc. Metabol..

[79] Jeukendrup A.E., Jentjens R. (2000). Oxidation of carbohydrate feedings during prolonged exercise: Current thoughts, guidelines and directions for future research. Sports Med..

[80] Murray R., Bartoli W.P., Eddy D.E., Horn M.K. (1997). Gastric emptying and plasma deuterium accumulation following ingestion of water and two carbohydrate-electrolyte beverages. Int. J. Sport Nutr..

[81] Murray R., Bartoli W., Stofan J., Horn M., Eddy D. (1999). A comparison of the gastric emptying characteristics of selected sports drinks. Int. J. Sport Nutr..

[82] Lundholm K., Edstrom S., Ekman L., Karlberg I., Walker P., Schersten T. (1981). Protein degradation in human skeletal muscle tissue: The effect of insulin, leucine, amino acids and ions. Clin. Sci..

[83] Nagasawa T., Kido T., Yoshizawa F., Ito Y., Nishizawa N. (2002). Rapid suppression of protein degradation in skeletal muscle after oral feeding of leucine in rats. J. Nutr. Biochem..

[84] Biolo G., Maggi S.P., Williams B.D., Tipton K.D., Wolfe R.R. (1995). Increased rates of muscle protein turnover and amino acid transport after resistance exercise in humans. Am. J. Physiol. Endocrinol. Metabol..

[85] Phillips S.M., Tipton K.D., Aarsland A., Wolf S.E., Wolfe R.R. (1997). Mixed muscle protein synthesis and breakdown after resistance exercise in humans. Am. J. Physiol. Endocrinol. Metabol..

[86] Levenhagen D.K., Carr C., Carlson M.G., Maron D.J., Borel M.J., Flakoll P.J. (2002). Postexercise protein intake enhances whole-body and leg protein accretion in humans. Med. Sci. Sports Exerc..

[87] Bohe J., Low A., Wolfe R.R., Rennie M.J. (2003). Human muscle protein synthesis is modulated by extracellular, not intramuscular amino acid availability: A dose-response study. J. Physiol..

[88] Gorissen S.H., Rémond D., Van Loon L.J. (2015). The muscle protein synthetic response to food ingestion. Meat Sci..

[89] Tipton K., Gurkin B., Matin S., Wolfe R. (1999). Nonessential amino acids are not necessary to stimulate net muscle protein synthesis in healthy volunteers. J. Nutr. Biochem..

[90] West D.W., Burd N.A., Coffey V.G., Baker S.K., Burke L.M., Hawley J.A., Moore D.R., Stellingwerff T., Phillips S.M. (2011). Rapid aminoacidemia enhances myofibrillar protein synthesis and anabolic intramuscular signaling responses after resistance exercise. Am. J. Clin. Nutr..

[91] Suryawan A., Jeyapalan A.S., Orellana R.A., Wilson F.A., Nguyen H.V., Davis T.A. (2008). Leucine stimulates protein synthesis in skeletal muscle of neonatal pigs by enhancing mTORC1 activation. Am. J. Physiol. Endocrinol. Metabol..

[92] Breen L., Phillips S.M. (2012). Nutrient interaction for optimal protein anabolism in resistance exercise. Curr. Opin. Clin. Nutr. Metab. Care.

[93] Zaromskyte G., Prokopidis K., Ioannidis T., Tipton K.D., Witard O.C. (2021). Evaluating the leucine trigger hypothesis to explain the post-prandial regulation of muscle protein synthesis in young and older adults: A systematic review. Front. Nutr..

[94] Elliot T.A., Cree M.G., Sanford A.P., Wolfe R.R., Tipton K.D. (2006). Milk ingestion stimulates net muscle protein synthesis following resistance exercise. Med. Sci. Sports Exerc..

[95] Wolfe R.R. (2000). Effects of insulin on muscle tissue. Curr. Opin. Clin. Nutr. Metab. Care..

[96] Biolo G., Williams B.D., Fleming R.Y., Wolfe R.R. (1999). Insulin action on muscle protein kinetics and amino acid transport during recovery after resistance exercise. Diabetes.

[97] Manninen A.H. (2006). Hyperinsulinaemia, hyperaminoacidaemia and post-exercise muscle anabolism: The search for the optimal recovery drink. Br. J. Sports Med..

[98] Mahe S., Roos N., Benamouzig R., Davin L., Luengo C., Gagnon L., Gausserges N., Rautureau J., Tome D. (1996). Gastrojejunal kinetics and the digestion of [15N]beta-lactoglobulin and casein in humans: The influence of the nature and quantity of the protein. Am. J. Clin. Nutr..

[99] Boirie Y., Dangin M., Gachon P., Vasson M.P., Maubois J.L., Beaufrere B. (1997). Slow and fast dietary proteins differently modulate postprandial protein accretion. Proc. Natl. Acad. Sci. USA.

[100] Dangin M., Boirie Y., Garcia-Rodenas C., Gachon P., Fauquant J., Callier P., Ballevre O., Beaufrere B. (2001). The digestion rate of protein is an independent regulating factor of postprandial protein retention. Am. J. Physiol. Endocrinol. Metabol..

[101] Dangin M., Boirie Y., Guillet C., Beaufrere B. (2002). Influence of the protein digestion rate on protein turnover in young and elderly subjects. J. Nutr..

[102] Ferrando A.A., Wolfe R.R., Hirsch K.R., Church D.D., Kviatkovsky S.A., Roberts M.D., Stout J.R., Gonzalez D.E., Sowinski R.J., Kreider R.B. (2023). International Society of Sports Nutrition Position Stand: Effects of essential amino acid supplementation on exercise and performance. J. Int. Soc. Sports Nutr..

[103] Biolo G., Fleming R.Y., Maggi S.P., Wolfe R.R. (1995). Transmembrane transport and intracellular kinetics of amino acids in human skeletal muscle. Am. J. Physiol. Endocrinol. Metabol..

[104] Drummond M.J., Glynn E.L., Fry C.S., Timmerman K.L., Volpi E., Rasmussen B.B. (2010). An increase in essential amino acid availability upregulates amino acid transporter expression in human skeletal muscle. Am. J. Physiol. Endocrinol. Metabol..

[105] Coburn J.W., Housh D.J., Housh T.J., Malek M.H., Beck T.W., Cramer J.T., Johnson G.O., Donlin P.E. (2006). Effects of leucine and whey protein supplementation during eight weeks of unilateral resistance training. J. Strength Cond. Res..

[106] Deldicque L., Theisen D., Francaux M. (2005). Regulation of mTOR by amino acids and resistance exercise in skeletal muscle. Eur. J. Appl. Physiol..

[107] Gibala M.J. (2000). Nutritional supplementation and resistance exercise: What is the evidence for enhanced skeletal muscle hypertrophy?. Can. J. Appl. Physiol..

[108] Gater D.R., Gater D.A., Uribe J.M., Bunt J.C. (1992). Impact of nutritional supplements on body composition, strength and IGF-1. J. Appl. Sport Sci. Res..

[109] Rozenek R., Ward P., Long S., Garhammer J. (2002). Effects of high-calorie supplements on body composition and muscular strength following resistance training. J. Sports Med. Phys. Fitness.

[110] Kreider R.B. (1999). Dietary supplements and the promotion of muscle growth with resistance exercise. Sports Med..

[111] Churchward-Venne T.A., Burd N.A., Phillips S.M. (2012). Nutritional regulation of muscle protein synthesis with resistance exercise: Strategies to enhance anabolism. Nutr. Metab..

[112] Atherton P.J., Smith K. (2012). Muscle protein synthesis in response to nutrition and exercise. J. Physiol..

[113] Hornberger T.A., Esser K.A. (2004). Mechanotransduction and the regulation of protein synthesis in skeletal muscle. Proc. Nutr. Soc..

[114] Hornberger T.A. (2011). Mechanotransduction and the regulation of mTORC1 signaling in skeletal muscle. Int. J. Biochem. Cell. Biol..

[115] Hornberger T.A., Chien S. (2006). Mechanical stimuli and nutrients regulate rapamycin-sensitive signaling through distinct mechanisms in skeletal muscle. J. Cell. Biochem..

[116] Huang J., Manning B.D. (2009). A complex interplay between Akt, TSC2 and the two mTOR complexes. Biochem. Soc. Trans..

[117] Navé B.T., Ouwens D.M., Withers D.J., Alessi D.R., Shepherd P.R. (1999). Mammalian target of rapamycin is a direct target for protein kinase B: Identification of a convergence point for opposing effects of insulin and amino-acid deficiency on protein translation. Biochem. J..

[118] Bolster D.R., Kubica N., Crozier S.J., Williamson D.L., Farrell P.A., Kimball S.R., Jefferson L.S. (2003). Immediate response of mammalian target of rapamycin (mtor)-mediated signalling following acute resistance exercise in rat skeletal muscle. J. Physiol..

[119] Kimball S.R., Farrell P.A., Jefferson L.S. (2002). Role of insulin in translational control of protein synthesis in skeletal muscle by amino acids or exercise. J. Appl. Physiol..

[120] DeYoung M.P., Horak P., Sofer A., Sgroi D., Ellisen L.W. (2008). Hypoxia regulates TSC1/2–mTOR signaling and tumor suppression through REDD1-mediated 14–3–3 shuttling. Genes. Dev..

[121] Caldow M.K., Thomas E.E., Dale M.J., Tomkinson G.R., Buckley J.D., Cameron-Smith D. (2015). Early myogenic responses to acute exercise before and after resistance training in young men. Physiol. Rep..

[122] Gibala M. (2009). Molecular responses to high-intensity interval exercise. Appl. Physiol. Nutr. Metab..

[123] Lundberg T.R., Fernandez-Gonzalo R., Gustafsson T., Tesch P.A. (2013). Aerobic exercise does not compromise muscle hypertrophy response to short-term resistance training. J. Appl. Physiol..

[124] Roberts M.D., McCarthy J.J., Hornberger T.A., Phillips S.M., Mackey A.L., Nader G.A., Boppart M.D., Kavazis A.N., Reidy P.T., Ogasawara R. (2023). Mechanisms of mechanical overload-induced skeletal muscle hypertrophy: Current understanding and future directions. Physiol. Rev..

[125] Franchi M.V., Atherton P.J., Reeves N.D., Flück M., Williams J., Mitchell W.K., Selby A., Beltran Valls R.M., Narici M.V. (2014). Architectural, functional and molecular responses to concentric and eccentric loading in human skeletal muscle. Acta Physiol..

[126] Lecker S.H., Goldberg A.L., Mitch W.E. (2006). Protein Degradation by the Ubiquitin–Proteasome Pathway in Normal and Disease States. J. Am. Soc. Nephrol..

[127] Borgenvik M., Apró W., Blomstrand E. (2012). Intake of branched-chain amino acids influences the levels of MAFbx mRNA and MuRF-1 total protein in resting and exercising human muscle. Am. J. Physiol. Endocrinol. Metabol..

[128] Mascher H., Tannerstedt J., Brink-Elfegoun T., Ekblom B., Gustafsson T., Blomstrand E. (2008). Repeated resistance exercise training induces different changes in mRNA expression of MAFbx and MuRF-1 in human skeletal muscle. Am. J. Physiol. Endocrinol. Metabol..

[129] Stefanetti R.J., Lamon S., Wallace M., Vendelbo M.H., Russell A.P., Vissing K. (2015). Regulation of ubiquitin proteasome pathway molecular markers in response to endurance and resistance exercise and training. Pflug. Arch. Eur. J. Physiol..

[130] Stitt T.N., Drujan D., Clarke B.A., Panaro F., Timofeyva Y., Kline W.O., Gonzalez M., Yancopoulos G.D., Glass D.J. (2004). The IGF-1/PI3K/Akt pathway prevents expression of muscle atrophy-induced ubiquitin ligases by inhibiting foxo transcription factors. Mol. Cell.

[131] Gulati P., Gaspers L.D., Dann S.G., Joaquin M., Nobukuni T., Natt F., Kozma S.C., Thomas A.P., Thomas G. (2008). Amino acids activate mtor complex 1 via Ca2+/CaM signaling to hVps34. Cell. Metab..

[132] Takahara T., Amemiya Y., Sugiyama R., Maki M., Shibata H. (2020). Amino acid-dependent control of mTORC1 signaling: A variety of regulatory modes. J. Biomed. Sci..

[133] Dreyer H.C., Fujita S., Cadenas J.G., Chinkes D.L., Volpi E., Rasmussen B.B. (2006). Resistance exercise increases AMPK activity and reduces 4E-BP1 phosphorylation and protein synthesis in human skeletal muscle. J. Physiol..

[134] Drummond M.J., Rasmussen B.B. (2008). Leucine-enriched nutrients and the regulation of mammalian target of rapamycin signalling and human skeletal muscle protein synthesis. Curr. Opin. Clin. Nutr. Metab. Care.

[135] Fujita S., Dreyer H.C., Drummond M.J., Glynn E.L., Cadenas J.G., Yoshizawa F., Volpi E., Rasmussen B.B. (2007). Nutrient signalling in the regulation of human muscle protein synthesis. J. Physiol..

[136] Atherton P.J., Etheridge T., Watt P.W., Wilkinson D., Selby A., Rankin D., Smith K., Rennie M.J. (2010). Muscle full effect after oral protein: Time-dependent concordance and discordance between human muscle protein synthesis and mTORC1 signaling1234. Am. J. Clin. Nutr..

[137] Areta J.L., Burke L.M., Camera D.M., West D.W.D., Crawshay S., Moore D.R., Stellingwerff T., Phillips S.M., Hawley J.A., Coffey V.G. (2014). Reduced resting skeletal muscle protein synthesis is rescued by resistance exercise and protein ingestion following short-term energy deficit. Am. J. Physiol. Endocrinol. Metabol..

[138] Karlsson H.K.R., Nilsson P.-A., Nilsson J., Chibalin A.V., Zierath J.R., Blomstrand E. (2004). Branched-chain amino acids increase p70S6k phosphorylation in human skeletal muscle after resistance exercise. Am. J. Physiol. Endocrinol. Metab..

[139] Apró W., Blomstrand E. (2010). Influence of supplementation with branched-chain amino acids in combination with resistance exercise on p70S6 kinase phosphorylation in resting and exercising human skeletal muscle. Acta Physiol..

[140] Lam F.-C., Khan T.M., Faidah H., Haseeb A., Khan A.H. (2019). Effectiveness of whey protein supplements on the serum levels of amino acid, creatinine kinase and myoglobin of athletes: A systematic review and meta-analysis. Syst. Rev..

[141] Farnfield M.M., Breen L., Carey K.A., Garnham A., Cameron-Smith D. (2012). Activation of mTOR signalling in young and old human skeletal muscle in response to combined resistance exercise and whey protein ingestion. Appl. Physiol. Nutr. Metab..

[142] Kakigi R., Yoshihara T., Ozaki H., Ogura Y., Ichinoseki-Sekine N., Kobayashi H., Naito H. (2014). Whey protein intake after resistance exercise activates mTOR signaling in a dose-dependent manner in human skeletal muscle. Eur. J. Appl. Physiol..

[143] Koopman R., Pennings B., Zorenc A.H.G., van Loon L.J.C. (2007). Protein ingestion further augments S6K1 phosphorylation in skeletal muscle following resistance type exercise in males. J. Nutr..

[144] Ferreira M.P., Li R., Cooke M., Kreider R.B., Willoughby D.S. (2014). Periexercise coingestion of branched-chain amino acids and carbohydrate in men does not preferentially augment resistance exercise–induced increases in phosphatidylinositol 3 kinase/protein kinase B–mammalian target of rapamycin pathway markers indicative of muscle protein synthesis. Nutr. Res..

[145] Burd N.A., Gorissen S.H., van Vliet S., Snijders T., van Loon L.J.C. (2015). Differences in postprandial protein handling after beef compared with milk ingestion during postexercise recovery: A randomized controlled trial12. Am. J. Clin. Nutr..

[146] Reidy P.T., Walker D.K., Dickinson J.M., Gundermann D.M., Drummond M.J., Timmerman K.L., Fry C.S., Borack M.S., Cope M.B., Mukherjea R. (2013). Protein blend ingestion following resistance exercise promotes human muscle protein synthesis. J. Nutr..

[147] Reidy P.T., Walker D.K., Dickinson J.M., Gundermann D.M., Drummond M.J., Timmerman K.L., Cope M.B., Mukherjea R., Jennings K., Volpi E. (2014). Soy-dairy protein blend and whey protein ingestion after resistance exercise increases amino acid transport and transporter expression in human skeletal muscle. J. Appl. Physiol..

[148] Blomstrand E. (2006). A role for branched-chain amino acids in reducing central fatigue. J. Nutr..

[149] Newsholme E. (1987). Amino acids, brain neurotransmitters and a functional link between muscle and brain that is important in sustained exercise. Adv. Myochem..

[150] Davis J.M., Alderson N.L., Welsh R.S. (2000). Serotonin and central nervous system fatigue: Nutritional considerations. Am. J. Clin. Nutr..

[151] Davis J.M. (1995). Carbohydrates, branched-chain amino acids, and endurances: The central fatigue hypothesis. Int. J. Sport Nutr. Exerc. Metab..

[152] Matsumoto K., Koba T., Hamada K., Tsujimoto H., Mitsuzono R. (2009). Branched-chain amino acid supplementation increases the lactate threshold during an incremental exercise test in trained individuals. J. Nutr. Sci. Vitaminol..

[153] Hormoznejad R., Zare Javid A., Mansoori A. (2019). Effect of BCAA supplementation on central fatigue, energy metabolism substrate and muscle damage to the exercise: A systematic review with meta-analysis. Sport Sci. Health.

[154] Martinho D.V., Nobari H., Faria A., Field A., Duarte D., Sarmento H. (2022). Oral branched-chain amino acids supplementation in athletes: A systematic review. Nutrients.

[155] Aragon A., Schoenfeld B. (2013). Nutrient timing revisited: Is there a post-exercise anabolic window?. J. Int. Soc. Sports Nutr..

[156] Kerksick C.M., Arent S., Schoenfeld B.J., Stout J.R., Campbell B., Wilborn C.D., Taylor L., Kalman D., Smith-Ryan A.E., Kreider R.B. (2017). International Society of Sports Nutrition Position Stand: Nutrient Timing. J. Int. Soc. Sports Nutr..

